# Ultrafast Diffusion of a Fluorescent Cholesterol Analog in Compartmentalized Plasma Membranes

**DOI:** 10.1111/tra.12163

**Published:** 2014-03-11

**Authors:** Nao Hiramoto-Yamaki, Kenji A K Tanaka, Kenichi G N Suzuki, Koichiro M Hirosawa, Manami S H Miyahara, Ziya Kalay, Koichiro Tanaka, Rinshi S Kasai, Akihiro Kusumi, Takahiro K Fujiwara

**Affiliations:** Institute for Integrated Cell-Material Sciences (WPI-iCeMS) and Institute for Frontier Medical Sciences, Kyoto UniversityKyoto, 606-8507, Japan

**Keywords:** cholesterol, hop diffusion, phospholipids, raft domains, single fluorescent-molecule tracking, single-particle tracking

## Abstract

Cholesterol distribution and dynamics in the plasma membrane (PM) are poorly understood. The recent development of Bodipy488-conjugated cholesterol molecule (Bdp-Chol) allowed us to study cholesterol behavior in the PM, using single fluorescent-molecule imaging. Surprisingly, in the intact PM, Bdp-Chol diffused at the fastest rate ever found for any molecules in the PM, with a median diffusion coefficient (*D*) of 3.4 µm^2^/second, which was ∼10 times greater than that of non-raft phospholipid molecules (0.33 µm^2^/second), despite Bdp-Chol's probable association with raft domains. Furthermore, Bdp-Chol exhibited no sign of entrapment in time scales longer than 0.5 milliseconds. In the blebbed PM, where actin filaments were largely depleted, Bdp-Chol and Cy3-conjugated dioleoylphosphatidylethanolamine (Cy3-DOPE) diffused at comparable *D*s (medians = 5.8 and 6.2 µm^2^/second, respectively), indicating that the actin-based membrane skeleton reduces the *D* of Bdp-Chol only by a factor of ∼2 from that in the blebbed PM, whereas it reduces the *D* of Cy3-DOPE by a factor of ∼20. These results are consistent with the previously proposed model, in which the PM is compartmentalized by the actin-based membrane-skeleton fence and its associated transmembrane picket proteins for the macroscopic diffusion of all of the membrane molecules, and suggest that the probability of Bdp-Chol passing through the compartment boundaries, once it enters the boundary, is ∼10× greater than that of Cy3-DOPE. Since the compartment sizes are greater than those of the putative raft domains, we conclude that raft domains coexist with membrane-skeleton-induced compartments and are contained within them.

Cholesterol is considered as a key molecule for plasma membrane (PM) organization 1. It is likely to form stoichiometric complexes with saturated alkyl chains 2,3 and to hydrogen bond with sphingomyelins and glycosphingolipids 4,5. Meanwhile, cholesterol is not readily miscible with unsaturated alkyl chains, due to the non-conformability between its rigid, bulky tetracyclic sterol backbone and the rigid, bent *cis*-double bonds of the unsaturated phospholipid 6,7. Therefore, in the presence of *saturated* alkyl chains in the membrane, cholesterol molecules would be segregated out of unsaturated lipid domains, and form (transient) complexes or domains with the saturated alkyl chains of glycosylphosphatidylinositol (GPI)-anchored proteins, glycosphingolipids and sphingomyelin (however, see 8). Due to these properties, cholesterol has been considered as an essential molecule for cooperative assemblies of various raft domains in the PM as well as in artificial membrane bilayers 9,10.

Despite the importance of cholesterol in the PM organization, particularly in raft domain formation, cholesterol dynamics in the cellular PM has hardly been investigated. This is mainly due to the lack of fluorescent cholesterol analogs that can be easily used for fluorescent microscopic examinations. Dehydroergosterol was used as a fluorescent analog in a living cell PM 11, but it was difficult to track, as it exhibited fast photobleaching and low fluorescence intensity due to an unfavorable excitation wavelength for fluorescence microscopy.

Recently, a Bodipy488-conjugated cholesterol molecule (Bdp-Chol, the dye is linked to carbon-24 of the sterol's alkyl side chain) was developed, which can be imaged at the single-molecule level in the PM 12,13. This probe has been shown to partition into the liquid-ordered domain in model membranes 13–15, to partition into the detergent-resistant membrane (DRM) in both human coronary artery smooth muscle (HASM) and COS-7 cells (Figure S1), and to behave similarly to cholesterol in both normal and cholesterol-storage disease cells 16. Furthermore, upon injection into the yolk sac, Bdp-Chol did not disturb zebrafish development and was targeted to sterol-enriched brain regions in live fish 16. By observing Bdp-Chol using fluorescence correlation spectroscopy (ensemble-averaged measurements, rather than single-molecule measurements), based on conventional confocal and stimulation-induced emission depletion confocal microscopy, Solanko et al. 17 found that Bdp-Chol in the PM of epithelial Vero cells undergoes simple-Brownian diffusion, without any sign of anomalous subdiffusion in space scales greater than 80 nm. However, this scale is greater than the spatial scale of interest in the PM, because subdiffusion is likely to occur in *space scales smaller than* the size of the PM compartments generated by the actin-based membrane skeleton and/or nanoscale raft domains. Therefore, further examination of cholesterol diffusion, using methods sensitive to subdiffusion on much smaller scales, should be conducted.

In artificial lipid membranes containing various concentrations of cholesterol, ^1^H-pulsed field-gradient magic-angle spinning NMR spectroscopy revealed that cholesterol undergoes rapid simple-Brownian diffusion with *diffusion rates comparable to or slightly greater than those for phospholipids* 18. Meanwhile, Solanko et al. 17 found that Bdp-Chol diffused substantially slower than phospholipids in artificial membranes (at diffusion coefficients [*D*] of 1.6–3.2 versus 5–10 µm^2^/second; 19). Surprisingly, *D* of Bdp-Chol in the intact PM of the Vero cells (1.7 µm^2^/second) is comparable to that in artificial membranes.

Therefore, the first objective of the present study was (i) to observe Bdp-Chol in the PMs of several cell lines by high-speed single-molecule imaging, which enables observations with spatial precisions of ∼20 nm at time resolutions of 0.5 milliseconds and 20 microseconds, and to compare the results with the phospholipid data.

We used three cell lines, PtK2 epithelial cells, COS-7 epithelial cells (often referred to as fibroblast-like cells), and human coronary artery smooth muscle (HASM) cells. The use of these three cell lines was, first of all, for examining the generality of the observed behavior the Bdp-Chol in the PMs. In selecting the three cell lines, we chose the cell lines that were previously considered, by other groups, to have PMs that differed from the PMs we had examined previously; i.e. the PMs that, according to previous studies by others, might not be partitioned by the actin-based membrane skeleton (at variance with our previous studies; 20).

PtK2 and COS-7 cells were employed because previous reports by Lenne et al. 21, using COS-7 cells, and Eggeling et al. 22 and Sahl et al. 23, employing PtK2 cells, stated that the raft-associated molecules are confined for ≥50% of the time in cholesterol-dependent microdomains (<120-nm and <20-nm domains in COS-7 and PtK2 cells, respectively) and diffusing (undergoing apparent simple-Brownian diffusion) at the same rate as non-raft molecules for the remaining ≤50% of the time. Meanwhile, Sezgin et al. 24 reported that some of the raft domains described in these advanced optical microscopic studies were indeed non-raft domains.

The second aim of this investigation was, thus, (ii) to examine whether cholesterol is trapped in nanoscale domains. In addition, since transient trapping should reduce the overall diffusion coefficient in longer time scales, we examined whether Bdp-Chol diffuses more slowly than non-raft phospholipids, such as a fluorescent dioleoylphosphatidylethanolamine (DOPE) probe.

The sizes of cholesterol-dependent microdomains have been reported to be <20 nm, <120 nm, and ∼700 nm in COS-7, PtK2 and HASM cells, respectively. Since the publication of our own review, which strongly advanced the argument of small raft domains, including those consisting of only three molecules 6, smaller raft domains of sizes of 5–20 nm have been reported 25–28. Meanwhile, the concept of large, stable rafts is still prevalent in the literature (29,30,31, reviewed by 7 32, 33), including even raft domains with sizes of several tens of microns 34. Note that the total areal fraction of raft domains might be as large as 25% in the PM 35, but each individual raft domain could be a few nanometers in diameter.

One of the most prominent and frequently cited papers about micron-scale rafts is that by Schütz et al. 36, who reported the prevalent existence of raft domains of 700 nm in diameter in HASM cells. Recently, the concept in which a fully saturated lipid is confined to half-micron-sized cholesterol-dependent domains in the HASM-cell PM has been strongly promoted 37. The literature is still quite confused with regard to the basic raft sizes in the PMs of steady-state, non-stimulated cells 38,39

Therefore, the third aim of this research was (iii) to examine whether 700-nm raft domains exist generally in other cell types or only in HASM cells. The HASM-cell PM might be special, due to the enriched presence of caveolae. Since caveolae tend to be abundant in smooth muscle cells (e.g. 35/µm^2^; 40, also see 41), we considered the possibility that the raft domains of the HASM-cell PM may be very different from those of other cell types. Furthermore, in the investigation reported by Schütz et al. 36, a lower observation temperature (room temperature), rather than 37°C, and a fluorescent phospholipid probe, Cy5-conjugated l-α-dimyristoylphosphatidylethanolamine (Cy5-DMPE, which was assumed to be raft-associated by the authors without justification) were employed. In light of recent results of micron-scale raft formation when actin-depleted PM vesicles were cooled to around 12°C 42–44, the possibility of the formation of micron-scale raft domains at lower temperatures, even in the presence of the actin-based membrane skeleton, is of particular interest. Therefore, in the present investigation, the dynamics of both phospholipids and Bdp-Chol in the HASM-cell PM was extensively investigated at both 37°C and 24°C, with special attention paid to the presence of half-micron-sized (700 nm in diameter) cholesterol-dependent domains that strongly confine fully saturated phospholipids.

Another key component for understanding molecular dynamics in the PM is its partitioning or compartmentalization 20,45–47. This occurs due to the presence of the actin-based membrane skeleton meshwork on the cytoplasmic surface of the PM (fence), and the transmembrane proteins anchored to the actin filaments, lining the membrane skeleton fence (pickets, acting on virtually all membrane molecules by steric hindrance and circumferential slowing effects). Even the phospholipids and GPI-anchored proteins located in the PM outer leaflet are temporarily confined in these compartments, due to the corralling effect of the pickets aligned along the membrane skeleton fence 45,48.

Therefore, the fourth purpose of the present study was (iv-a) to investigate whether the PM partitioning-compartmentalization by the actin-based membrane skeleton exists in the HASM-cell PM, which might be very different from the PMs of other cell types; (iv-b) to determine, if the PM partitioning-compartmentalization exists in the HASM-cell PM, the compartment size and residency time of phospholipids; and (iv-c) to examine how effectively the PM partitioning affects Bdp-Chol mobility in the PM and to obtain the residency time of Bdp-Chol within a compartment. The PM partitioning is known to slow the diffusion of phospholipids, GPI-anchored proteins and TM proteins in the PM by a factor of ∼20, as compared with the PM in the absence of its associated actin filaments (in the blebbed PM, which might further be treated with actin depolymerization drugs) 20,47, and we are particularly interested in the extent to which Bdp-Chol diffusion is slowed by the PM fence-pickets.

Finally, the fifth objective of the present study was (v) to understand how the PM partitioning, induced by the actin-based membrane skeleton and its associated picket proteins, and the cholesterol-enriched raft domains, induced by their molecular (im)miscibilities within the bilayer, coexist in the PM, based on the results obtained by accomplishing aims (i)–(iv). We think this is one of the critical questions for understanding molecular trafficking and signaling in the PM. One of our hypotheses is that the raft domains might generally be included within actin-induced compartments and/or fill some compartments, but rarely extend over the compartment boundaries 7.

## Results

### Cold-Triton solubilities of Bdp-Chol and other phospholipid probes incorporated in the PM

The insolubility of a PM molecule upon cold-Triton treatment has often been associated with its partitioning into raft domains in the live-cell PM, although this has never been proved (6 7, 20; reviewed in 32). Therefore, in this research, we examined the cold-Triton solubilities of Bdp-Chol and other phospholipid probes incorporated in the PM, for comparisons with those of other prototypical raft and non-raft molecules in the literature, such as GPI-anchored proteins (e.g. CD59, Thy1 and DAF) and transferrin receptor, respectively. The HASM and COS-7 PMs that incorporated Bdp-Chol, fluorescent phospholipid probes, [Cy3-conjugated phosphatidylethanolamines (Cy3-PEs)], collectively representing Cy3-dioleoyl-PE, Cy3-dimyristoyl-PE and Cy3-dipalmitoyl-PE, abbreviated as Cy3-DOPE, Cy3-DMPE and Cy3-DPPE, respectively) or Bodipy FL (Life Technologies)-conjugated GM1 in its alkyl chain (Bdpø-GM1), were extracted with cold Triton (1%, at 2.8°C for 15 min; Figure S1). Bdp-Chol, as well as the prototypical raft-associated molecular complex of the endogenous ganglioside GM1 and Cy3-conjugated cholera toxin B subunit, mostly remained in the DRMs. In contrast, all three species of Cy3-PEs and Bdpø-GM1 were almost entirely solubilized; i.e. Cy3-DMPE, Cy3-DPPE and Bdpø-GM1 are cold-Triton soluble, like the prototypical non-raft molecule Cy3-DOPE. Cy5-DMPE 36 and Bdpø-GM1 21 were previously assumed to be raft-associated without any experimental data, but the results obtained here indicate that these molecules are not prototypical DRM-associated molecules. The present results are consistent with the data obtained by Sezgin et al. 24. Meanwhile, the partitioning of Bdp-Chol in DRM suggested that Bdp-Chol could be a good analogue for native cholesterol, consistent with previous data (see *Introduction*).

### Single-molecule imaging and tracking of Bdp-Chol and other phospholipid probes in the top PM: their locations in the PM

Bdp-Chol and Cy3-PE molecules incorporated in the top PM of the cultured cells (the PM facing the buffer, rather than the coverslip) for less than 5 min were observed at the single-molecule level, using oblique-angle (HILO) illumination 49 based on a home-built objective-lens-type total internal reflection fluorescence (TIRF) microscope, at time resolutions of 0.5 and 33 milliseconds 50. Single-molecule imaging and tracking of many molecules simultaneously at a time resolution of 0.5 milliseconds was, to the best of our knowledge, the fastest single-molecule observations ever performed. The bottom PM (the PM facing the coverslip) was not observed, because it was difficult to differentiate the membrane-incorporated probes from those bound to the coverslip (exhibiting temporary binding and sudden hops to locations nearby) and from those internalized by endocytosis (these endosomes often remained near the bottom PM, exhibiting rapid movements along the bottom PM with intermittent cessations of diffusion; in contrast, the endosomes formed near the top PM tended to disappear rapidly from the focal plane). The observed fluorescent spots were photobleached in a single step, suggesting that single molecules of Bdp-Chol were observed. Even if cholesterol forms concentrated domains or oligomers, under our experimental conditions, in which very low concentrations of Bdp-Chol are incorporated in the PM (so that ∼1000 fluorescent spots/cell are visible), containing large amounts of endogenous cholesterol (more than 10^9^ molecules/cell), the domains and oligomers would not have been detectable. For observations of a typical non-raft control molecule, the unsaturated phospholipid Cy3-DOPE was incorporated in the PM at similar concentrations.

Several reports indicated that cholesterol is asymmetrically distributed between the outer and inner leaflets in the PM 11,51,52, and that 60–80% of the cholesterol in the PM partitions into the inner leaflet 11,52. Therefore, Bdp-Chol is likely to be located in both leaflets, possibly with higher partitioning into the inner leaflet. To investigate the Bdp-Chol distribution in the two leaflets, we examined the quenching of Bdp-Chol fluorescence in HASM cells, after the addition of Cu(II)meso-tetra(4-sulfonatophenyl)porphine (CuTSP, Figure S2A). Since separate control experiments indicated that fluorescence quenching by CuTSP strongly depends on the dyes' fluorescence spectra, the quenching is considered to occur due to Förster resonance energy transfer (FRET). From the fluorescence emission spectrum of the Bodipy488 moiety of Bdp-Chol and the absorption spectrum of CuTSP, the Förster distance was determined to be 5.1 nm.

CuTSP is water-soluble and thus expected to diffuse slowly across the PM. The fluorescent signal from Bdp-conjugated DPPE in its headgroup (Bdp-DPPE, Figure S2A), incorporated in the PM, completely disappeared immediately after the addition of 3.3 mm CuTSP (Figure S2B). This result showed that (i) CuTSP is suitable to quench Bdp fluorescence, (ii) it has access to the membrane surface, and (iii) the flipping of Bdp-DPPE is slow (much slower than 15 min, the maximal incubation + observation duration). When R110, which has an excitation spectrum very similar to that of Bodipy488, was localized on the PM cytoplasmic surface by using Lyn-kinase with the halo-tag protein and R110-labeled halo ligand (Bodipy488-linked halo ligand could not be used to label Lyn-halo protein because this compound is not membrane permeable), the quenching of R110 fluorescence by CuTSP was limited to only ≤10%. This result indicated that although a Förster distance of 5.1 nm is comparable to the PM thickness, 3.3 mm CuTSP added to the extracellular medium was not sufficient to quench the fluorescence of the R110 moiety (and thus that of the Bdp moiety) located on the PM cytoplasmic surface (unpublished data).

The addition of 3.3 mm CuTSP to Bdp-Chol-labeled cells instantaneously reduced the fluorescence intensity, as fast as the reduction of Bdp-DPPE intensity, but not to zero, leaving 34 (± 11)% of the original signal (Figure S2B). The remaining signal was unchanged for 5 min after the quencher addition (longer incubations induced cell morphology changes, which prevented longer observations). Although simple interpretations of this result are not possible, it indicated that the Bdp moieties of some Bdp-Chol molecules are protected from quenching by CuTSP on/near the extracellular surface.

Even if all of the Bdp-Chol molecules are located in the outer leaflet, because the Bdp group is likely to be located in the membrane interior, the signal level might only decrease to 34%. However, Mondal et al. 11 reported that 80% of dehydroergosterol is located in the inner leaflet, when it is incorporated in the PM. Therefore, if Bdp-Chol is distributed in a similar manner to dehydroergosterol, then the non-quenched fraction might largely represent the Bdp-Chol molecules located in the inner leaflet.

Meanwhile, Oren et al. 53 theoretically derived the favorable locations, along the membrane normal, of steroid hormone molecules that contain two polar hydroxyl and ketone groups at both ends of the long axis of the four rigid, planar, fused rings of the sterol backbone. They indicated the possibilities in which (i) either one of the polar groups is placed at the interface between the membrane and the aqueous phase (see Figure 5 in 53) and (ii) the second preferred location is in the middle of the bilayer, where the steroid hormone molecule places both polar end groups relatively closer to the both surfaces of the membrane (see Figure 6 in 53). We would not favor the Bdp-Chol orientation with the Bdp moiety on the membrane surface, due to its bulkiness. The Bdp group could be placed more readily in the membrane interior, where such a bulky group could be more easily accommodated due to the alkyl chain flexibility (Figure S2C). However, we consider the second preferred location found by Oren et al. 53 quite possible for the location of Bdp-Chol (Figure S2C). Indeed, using a molecular dynamics simulation, Khelashvili et al. 54 found that the distribution of the location of the cholesterol's OH polar group in the depth direction of the membrane (along the bilayer normal) is spread ∼1 nm or around 30% of the bilayer hydrophobic thickness (see Figure 7 in 54), suggesting that cholesterol could penetrate quite deeply toward the bilayer center. This tendency could be enhanced in the presence of two polar groups at both ends of the sterol's long axis.

Taken together, we propose that Bdp-Chol is located in both bilayer leaflets, with two likely positions for each leaflet; i.e. four likely locations altogether (in addition to the locations shown in Figure S2C, the symmetric locations in the inner leaflet should be considered). The CuTSP quenching by 66% would include its effect on the Bdp moiety located in these different locations. The discussions in the following parts of this report will assume such distributions of Bdp-Chol locations in the membrane.

### Analysis of single-molecule diffusion of Bdp-Chol and Cy3-DOPE in the top PM

**Figure 1:**
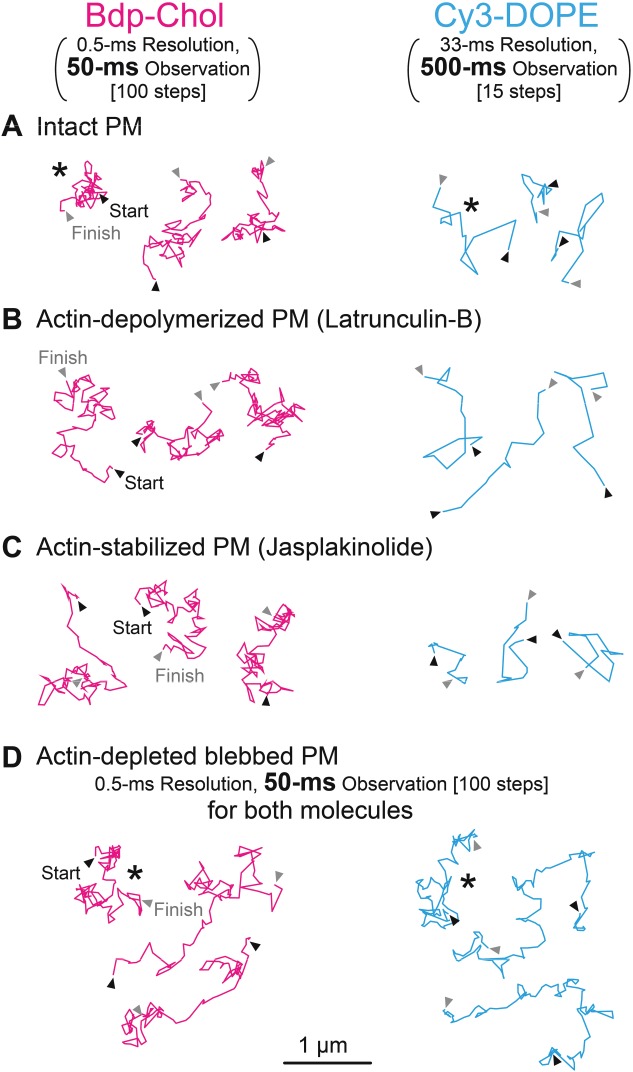
Representative Bdp-Chol (magenta) and Cy3-DOPE (blue) trajectories, obtained by single fluorescent-molecule tracking in the intact and variously treated PMs of PtK2 cells. All Bdp-Chol trajectories (magenta) were obtained at a 0.5-millisecond resolution for 50 milliseconds (100 points). The Cy3-DOPE trajectories (blue) were obtained at a 33-millisecond resolution for 500 milliseconds (15 points) except for those in actin-depleted blebbed PM (D), which were observed in exactly the same way as the Bdp-Chol trajectories. Arrowheads indicate the start (black) and finish (gray) points. The observation period of the Bdp-Chol shown here is 10 times shorter than that of Cy3-DOPE (A, B, C), to display the trajectories with very different diffusion coefficients on the same scale (similar space size).

Using the PtK2 cell line, which was previously employed by Eggeling et al. 22 and Sahl et al. 23, we examined the behaviors of Bdp-Chol and Cy3-DOPE in the top PM. Their typical trajectories are shown in Figure 1. A clearly remarkable feature of the trajectories in the intact cells, shown in Figure 1A, is that the tracking of a single Bdp-Chol molecule in the intact PM required an enhanced time resolution, from 33 milliseconds to 0.5 milliseconds, and that to place the typical trajectories of both Bdp-Chol and Cy3-DOPE in similar sizes, the trajectory lengths had to be different; i.e. 50- and 500-millisecond observation durations (at 0.5- and 33-millisecond resolutions for 100 and 15 steps, respectively). This clearly shows that Bdp-Chol diffused much faster than Cy3-DOPE.

**Figure 2:**
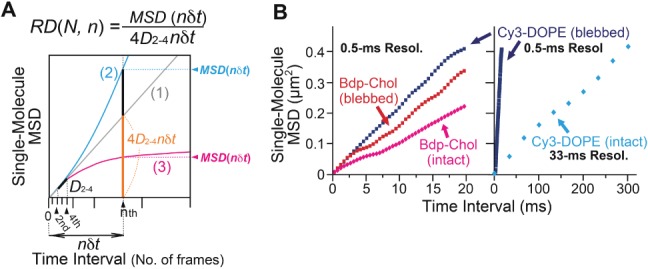
Diffusion mode classification based on the single-molecule *MSD-Δt* plot. A) Theoretical *MSD-Δt* plots for the three diffusion modes. The graphs were drawn assuming that the short-term diffusion coefficients (1/4 of the initial slope at *nδt* = 0) are the same for all of the modes. For the definitions of the parameters, see the text. With an increase in the deviation of *RD* from 1, the chances that the molecule undergoes directed (>1) or confined-hop (<1) diffusion increase. B) Typical single-molecule *MSD-Δt* plots for Bdp-Chol and Cy3-DOPE in the intact (Bdp-Chol, magenta; Cy3-DOPE, blue) or blebbed (Bdp-Chol, red; Cy3-DOPE, navy-blue) PMs of PtK2 cells. Each plot was obtained from the trajectory marked with an asterisk (*) in Figure 1A,B.

A single-molecule trajectory can be conveniently analyzed, by plotting the mean-square displacement (*MSD*) obtained from the trajectory against the time interval (*Δt*), called the single-molecule *MSD-Δt* plot (Figure 2A,B; 55). Each trajectory is classified into the (i) simple-Brownian, (ii) directed or (iii) suppressed diffusion mode, using the parameter *RD*(*N*, *n*), obtained from the *MSD-Δt* plot. *RD*(*N*, *n*) describes the relative deviation (*RD*) of the long-term diffusion of the molecule (*MSD*(*nδt*)) from the simple-Brownian model (expected from the short-term diffusion coefficient, 4*D*_2-4_*nδt*); i.e. *RD*(*n*) = *MSD*(*nδt*)/[4*D*_2-4_*nδt*], where *n* represents the number of steps used for the analysis in the trajectory of *N* steps, and *δt* represents the duration for each camera frame (0.5 or 33 milliseconds; 56). Therefore, *nδt* (*n* steps times the camera frame time *δt*) provides the time scale used for the mode classification of each trajectory. For non-Brownian motion, this *nδt* time scale used for the analysis is important, and therefore, in this report, we indicate this time scale when necessary (particularly, see the *x*-axes of Figure 2A,B).

**Figure 3:**
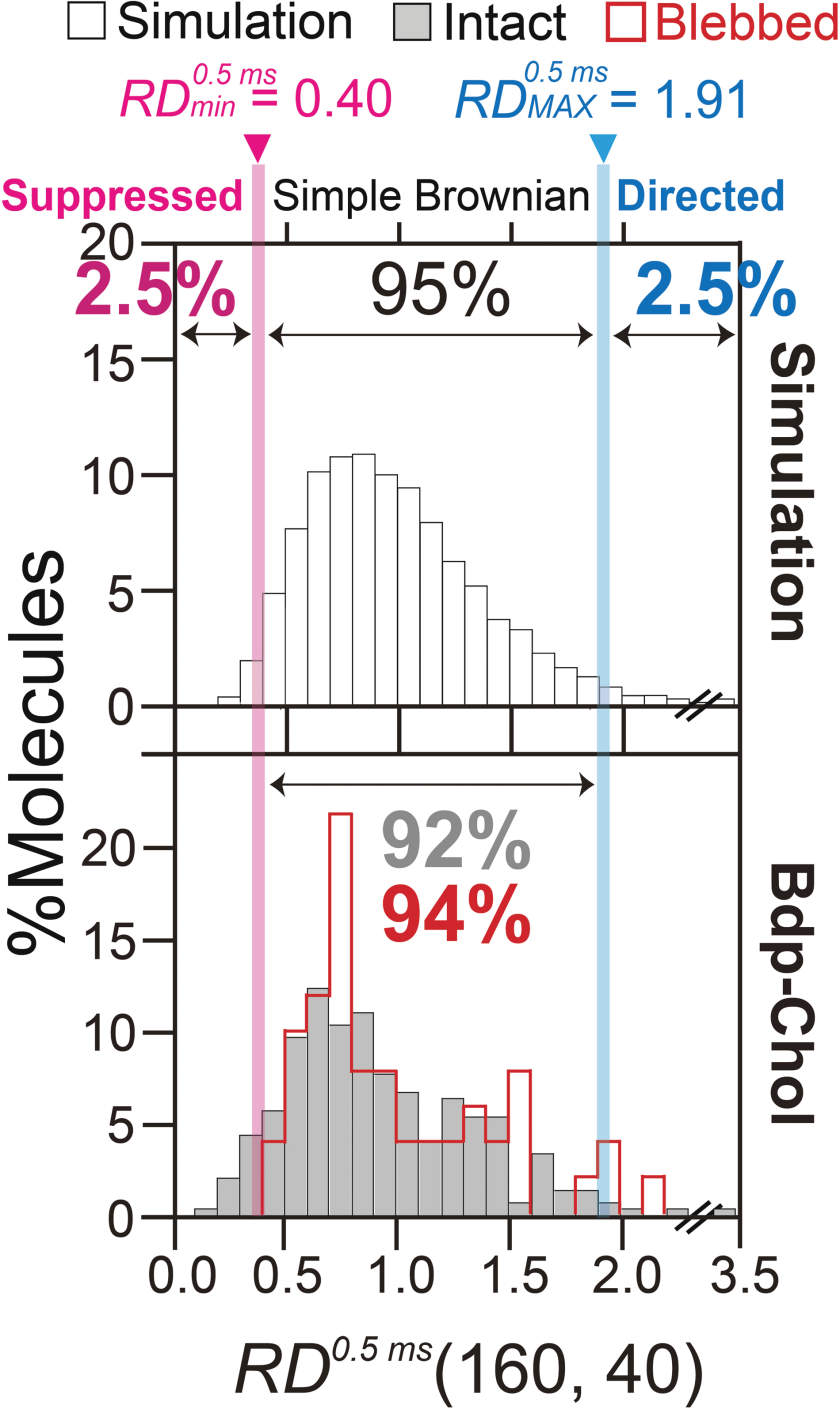
Bdp-Chol undergoes simple-Brownian diffusion in both the intact and blebbed PMs in the time scale of 20 milliseconds observed at a time resolution of 0.5 milliseconds. The distribution of the relative deviation *RD*(*N*, *n*) for *N* = 160, *n* = 40, and *δt* = 0.5 milliseconds, for each Bdp-Chol trajectory in intact (gray) and blebbed (red) PMs of PtK2 cells. The distribution of *RD*^0.5ms^(*N, n*) (the superscript indicates the time resolution of the recorded trajectories) for simple-Brownian particles was determined by using 10 000 simple-Brownian trajectories, generated by a Monte Carlo simulation (top). The *RD*^0.5ms^(*N, n*) values that gave the 2.5 percentiles of the particles from both ends of the distribution, referred to as *RD_min_* and *RD_MAX_*, were obtained (magenta and blue vertical lines, respectively). Each experimental single-molecule trajectory was classified into the suppressed-diffusion mode if its *RD*(*N, n*) value was smaller than *RD_min_*, into the directed-diffusion mode if its *RD*(*N, n*) value was larger than *RD_MAX_*, and into the simple-Brownian diffusion mode if *RD_min_* < *RD*(*N, n*) < *RD_MAX_* (therefore, in this analysis design, 95% of the ideal simple-Brownian particles should be classified into the simple-Brownian diffusion mode). Virtually all of the Bdp-Chol molecules were classified into the simple-Brownian diffusion mode, in both the intact and blebbed PMs at a 0.5-millisecond resolution. The numbers of trajectories examined are listed in Table1.

Molecules undergoing simple-Brownian diffusion show an average *RD*(*N*, *n*) of 1 (Figure 2A(1)), whereas molecules undergoing directed or suppressed diffusion exhibit averaged *RD*(*N*, *n*) values larger or smaller than 1, respectively (Figure 2A(2) and (3)). Since membrane molecules undergo thermal diffusion, the *RD*(*N*, *n*) value *for each individual trajectory* varies greatly from trajectory to trajectory. For example, although the average *RD*(*N*, *n*) is 1 for molecules undergoing simple-Brownian diffusion, the *RD*(*N*, *n*) value for each trajectory differs from 1. The *RD*(*N*, *n*) distribution for simulated simple-Brownian particles is shown in Figure 3 (*top*, open bars, ‘Simulation’). On the basis of this distribution, the *RD*(*N*, *n*) values giving the 2.5 percentiles of the particles from both ends of the distribution, referred to as *RD_min_* and *RD_MAX_*, were obtained. Each experimental single-molecule trajectory was classified into the suppressed-diffusion mode if its *RD*(*N*, *n*) value was smaller than *RD_min_*, and into the directed-diffusion mode if its *RD*(*N*, *n*) value was larger than *RD_MAX_*. Note that this classification is strictly based on the statistical deviations from simple-Brownian diffusion, and no diffusion model is assumed. To emphasize this point, we employ the term ‘suppressed diffusion’ here, rather than the ‘confined-hop diffusion’ term used in previous publications 45,48,56,57. Previously, since all of the *MSD-Δt* plots for the trajectories classified into the suppressed-diffusion mode could be fitted with the equation describing hop diffusion 58, the term ‘confined-hop diffusion’ was used for the particles exhibiting the ‘suppressed-diffusion mode’.

The *RD* distributions for Bdp-Chol obtained at a time resolution of 0.5 milliseconds (*δt*), in the time scale of 20 milliseconds (*nδt*, *n* = 40) in the intact PM, are shown in Figure 3 (also see Figure 2B left). Virtually all of the Bdp-Chol trajectories in the intact PM were categorized into the simple-Brownian diffusion mode in this time scale (Table1).

**Table 1:** The fractions of trajectories classified into the simple-Brownian diffusion mode and the *D^eff^_MACRO_* values of Bdp-Chol and Cy3-DOPE in intact and variously treated PMs

Lipid	Cell	Conditions	*D^eff^_MACRO_* (µm^2^/second)^a^	% Simple-Brownian^b^	*N*^c^
Cy3-DOPE	PtK2	Intact PM	0.32 ± 0.02	82	163
	PtK2	Actin-depleted blebbed PM	6.4 ± 0.01^*^^d^	96	299
	PtK2	Partial actin depolymerization	0.43 ± 0.03^*^	85	61
	PtK2	Actin stabilization	0.23 ± 0.02^*^	72	110
	PtK2	Partial cholesterol depletion	0.21 ± 0.01^*^	83	193
Bdp-Chol	PtK2	Intact PM	3.3 ± 0.08	92	298
	PtK2	Actin-depleted blebbed PM	6.3 ± 0.3^*^	94	51
	PtK2	Partial actin depolymerization	3.9 ± 0.1^*^	94	166
	PtK2	Actin stabilization	3.5 ± 0.2	82	134
	PtK2	Partial cholesterol depletion	3.0 ± 0.1^*^	79	145
	COS-7	Intact PM	2.8 ± 0.01	80	164
	HASM	Intact PM	3.3 ± 0.1	85	364

a Mean ± SE.

b Fractions of trajectories classified into the simple-Brownian diffusion mode, in the time scales of ≥20 milliseconds (Bdp-Chol under all of the conditions and Cy3-DOPE in the blebbed PM) and ≥1 second (Cy3-DOPE under all of the conditions except for the observations in the blebbed PM).

c Number of examined trajectories.

d Asterisks indicate statistically significant difference against the values found in the intact PM.

The same analysis was performed for Cy3-DOPE in the intact and blebbed PMs (in the time scales [*nδt*] of 1 second and 20 milliseconds, respectively), and again practically all of the Cy3-DOPE trajectories were classified into the simple-Brownian diffusion mode (Table1; also see Figure 2B right). Therefore, their diffusion can be characterized by *a single effective diffusion coefficient* (effective, because the observations made here is valid only in the indicated time scales; in most studies done by others, the time scales of the measurements are rarely shown, causing enormous confusions in the literature); i.e. *D^eff^_MACRO_*, which was determined by linear fitting between the second and fourth points in the *MSD-Δt* plot (*D*_2-4_ in Figure 2A; the reason for the subscript ‘MACRO’ will be explained later; 56). Time-lapse experiments covering time scales 10 times longer than those employed here (200 milliseconds and 10 seconds) also revealed that, in these time scales, virtually all of the trajectories are classified into the simple-Brownian diffusion mode. Therefore, rather than specifying the time scales as 20 milliseconds and 1 second, in the rest of this report, we will define the time scales as ‘≥20 milliseconds’ and ‘≥1 second’.

Similar analysis using *RD* distributions was carried out for Bdp-Chol and Cy3-DOPE trajectories obtained in the blebbed PMs (Figures 2B and 3 and Table1). The blebbed PM is a balloon-like structure formed from the PM, in which actin filaments are largely depleted, and in this examination, the actin filaments that may have remained on the cytoplasmic surface of the blebbed PM were further depleted, by a treatment with 10 µm cytochalasin D for 1 h. In the following part of this report, the actin-depleted blebbed PM prepared in this manner is simply referred to as the ‘blebbed PM’. Naturally, practically all of the Bdp-Chol and Cy3-DOPE trajectories obtained in blebbed PMs were categorized into the simple-Brownian diffusion mode in the time scales of ≥20 milliseconds (Table1).

### Bdp-Chol diffuses approximately 10-fold faster than Cy3-DOPE in the intact PM of PtK2 cells, although they both diffused quickly and at similar rates in the actin-depleted blebbed PM

In the following part of the main text, when we cite parameter values, we employ the mean values listed in tables, whereas we show median values of the distributions in histograms (some of the median values are also listed in tables wherever appropriate). When we compare two related values or discuss %-increases/reductions, we use these mean values, unless otherwise specified.

**Figure 4:**
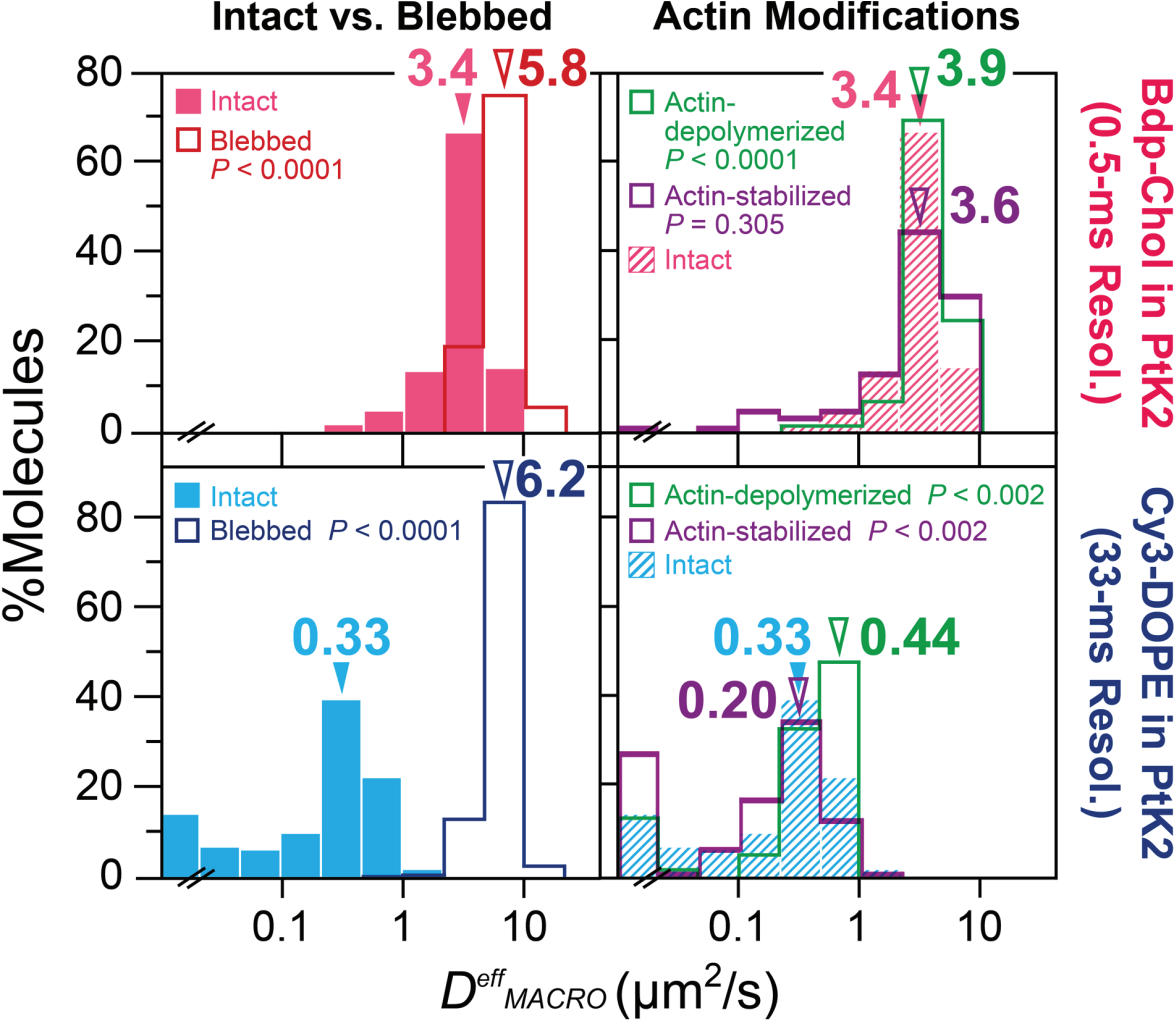
In the PtK2-cell PM, Bdp-Chol diffused ∼10× faster than Cy3-DOPE in the intact PM, whereas they diffused at about the same rate in the blebbed PM. In the intact PM, Bdp-Chol and Cy3-DOPE diffusion was slowed by factors of ∼2 and ∼20, respectively, from that in the blebbed PM, due to the actin-based membrane skeleton. The distribution of the effective diffusion coefficient, *D^eff^_MACRO_*, for each trajectory of Bdp-Chol (top) or Cy3-DOPE (bottom) in the intact PM (both left and right), after partial actin-depolymerization (right), after actin stabilization (right) and in the blebbed PM (left) of PtK2 cells. Arrowheads indicate median values. p values indicate the Mann–Whitney *U*-test results against the results in the intact PM. See the summary in Table1 (including the numbers of trajectories inspected).

The *D^eff^_MACRO_* values obtained in the intact and actin-depleted blebbed PMs of PtK2 cells are compared in Figure 4 (*left column*) and Table1. Note that since both Bdp-Chol and Cy3-DOPE diffused rapidly in the blebbed PM, both molecules were observed at a 0.5-millisecond resolution (typical trajectories are shown in Figure 1D; Bdp-Chol in the intact PM was observed at a 0.5-millisecond resolution for the same reason, but Cy3-DOPE in the intact PM was observed at a 33-millisecond resolution, due to its slow diffusion). Analyses of the distributions of *D^eff^_MACRO_* in the intact and blebbed PMs of PtK2 cells (Figure 4, *left column*, Table1) revealed that (i) in the blebbed PM, the *D^eff^_MACRO_* distributions of Bdp-Chol and Cy3-DOPE were very similar to each other (6.3 and 6.4 µm^2^/second, respectively), consistent with the data shown in Figures 1D and 2B; (ii) in the intact PM, *D^eff^_MACRO_* for Cy3-DOPE was reduced by a factor of ∼20 from that in the blebbed PM (0.32 versus 6.4 µm^2^/second), whereas *D^eff^_MACRO_* for Bdp-Chol was reduced only by a factor of ∼2 (3.3 versus 6.3 µm^2^/second) (consistent with the data shown in Figures 1A and 2B). (iii) As a result, *D^eff^_MACRO_* for Bdp-Chol in the intact PM was greater than that for Cy3-DOPE, by a factor of ∼10. To the best of our knowledge, a *D^eff^_MACRO_* value of 3.3 µm^2^/second is indeed the largest macroscopic diffusion coefficient of any molecules ever found in the intact PM 19. Note that this rapid Bdp-Chol diffusion coefficient does not mean that it diffuses quickly in the lipid membrane. In the blebbed PM, Bdp-Chol diffused at a rate similar to that of Cy3-DOPE, and also in artificial lipid bilayers, Bdp-Chol diffused at a rate comparable to those of phospholipids 15,59. Namely, the much larger diffusion coefficient of Bdp-Chol, as compared to that of Cy3-DOPE (∼10-fold) in the intact PM, is not due to the greater intrinsic diffusion coefficient of Bdp-Chol in the bilayer part of the bilayer. Instead, it is probably caused by the actin filaments associated with the PM, which were removed in the blebbed membrane. The hop probability of Bdp-Chol across the compartment boundaries, once it enters the boundary region, would be ∼10-fold greater than that of Cy3-DOPE.

It is possible that the transmembrane distributions of the probes are different in intact versus blebbed PMs. However, since the *D^eff^_MACRO_* distributions are sharp in the blebbed PM (Figure 4), *D^eff^_MACRO_* will be very similar in both the outer and inner leaflets (true for both Bdp-Chol and Cy3-DOPE). Therefore, the increase in *D^eff^_MACRO_* after f-actin depletion from the membrane in the blebbed PM would not be due to the possible changes in the transmembrane distributions of the probes. The appropriateness of using the blebbed PM as a control membrane, in which the actin-based membrane skeleton is depleted, will be discussed further in the *Discussion* section.

Such fast diffusion of Bdp-Chol in the intact PM is surprising, considering that it basically behaves like a raft-associated molecule (like cholesterol, including the property of partitioning into the DRM) because, if it were trapped in raft domains, then its diffusion would be slowed. In addition, we detected no sign of even temporary trapping in nanoscale domains in time scales longer than 0.5 milliseconds, using computer programs to detect transient arrest of lateral diffusion 60–62, including the presence of transient confinement zones 63 or transient immobilization-entrapment 23,64,65. This point is further clarified in the later subsection *Bdp-Chol is not confined in stand-alone microdomains (raft-like microdomains) detectable at a time resolution of 0.5 milliseconds, in the PtK2 PM*.

### Effects of partial depolymerization and stabilization of actin filaments on *D^eff^_MACRO_* of Bdp-Chol and Cy3-DOPE in the PtK2 PM

Previously, we found that the PtK2-cell PM was compartmentalized by the actin-based membrane skeleton and its associated transmembrane picket proteins lining the membrane skeleton, and that phospholipids undergo hop diffusion between the compartments 19. Here, the involvement of actin filaments in slowing the diffusion of Cy3-DOPE and Bdp-Chol in the intact PM was further examined, by using drugs that induce partial depolymerization and stabilization of actin filaments; i.e. latrunculin-B 45,66 and jasplakinolide 45,67, respectively (typical trajectories in Figure 1B and 1C, respectively). In the present research, the drug treatment conditions were finely tuned (see *Materials and Methods* for details).

#### Effects on D^eff^_MACRO_ of Cy3-DOPE in the PtK2 PM

The *D^eff^_MACRO_* values of Cy3-DOPE were significantly increased and decreased by 30%, after the cells were treated with 5 µm latrunculin-B and 25 µm jasplakinolide, respectively (Figure 4, *bottom right*; Table1), consistent with our previous observations in other cell types 19,45,48. These drug concentrations were much higher than those used previously, but PtK2 cells were extremely resistant to these drugs, and did not exhibit any morphological changes even under these conditions. Considering these results, along with the result showing a 20-fold increase of the Cy3-DOPE *D^eff^_MACRO_* value upon blebbing + actin depletion (Table1), we concluded that the actin filaments associated with the PM cytoplasmic surface are responsible for slowing the diffusion of Cy3-DOPE in the PtK2 PM, as found previously in other cell types.

#### Effects on D^eff^_MACRO_ of Bdp-Chol in the PtK2 PM

Under the same treatment conditions with latrunculin-B and jasplakinolide as employed for Cy3-DOPE observations described in the previous paragraph, the *D^eff^_MACRO_* values of Bdp-Chol increased by 18% (statistically significant) and 6% (non-significant), respectively (Figure 4, *top right*; Table1). Considering these results, together with the ∼2-fold increase of *D^eff^_MACRO_* of Bdp-Chol upon blebbing + actin depletion, we propose that the actin filaments associated with the PM cytoplasmic surface are responsible for slowing Bdp-Chol diffusion, in a similar manner to slowing Cy3-DOPE diffusion, despite our failure to detect the jasplakinolide effect. We will revisit this point later when we discuss similar experiments using the HASM-cell PM.

### Effects of cholesterol depletion on *D^eff^_MACRO_* values of Bdp-Chol and Cy3-DOPE in the PtK2 PM

Partial cholesterol depletion (using methyl-β-cyclodextrin, MβCD; 60 61, 68) significantly *reduced* (rather than increased) the *D^eff^_MACRO_* values of both Cy3-DOPE and Bdp-Chol, by 34 and 9.1%, respectively (Table1). These results are consistent with most of the previous findings, in which partial cholesterol depletion generally did not increase the *D^eff^_MACRO_* values of transmembrane and GPI-anchored proteins as well as cytoplasmic lipid-anchored molecules 48,69–71, and the diffusion of raft molecules was not slower than that of non-raft molecules 69. Previously (until the report by Kenworthy et al. 69), these results were considered to be at variance with the general concept that raft-associated molecules diffuse more slowly, as compared to non-raft-associated molecules.

Therefore, we can conclude that cholesterol-enriched raft domains do not reduce the macroscopic diffusion rates of either raft-associated (like Bdp-Chol) or non-raft-associated (like Cy3-DOPE) molecules in the PtK2-cell PM.

### Bdp-Chol is not confined in stand-alone microdomains (raft-like microdomains) detectable at a time resolution of 0.5 milliseconds, in the PtK2 PM

Sahl et al. 23 previously reported that their observations, at an enhanced time resolution of 0.5 milliseconds, revealed the temporary confinement of raft-associated molecules within microdomains more than 50% of the time, in the PM of PtK2 cells. Therefore, we examined the possibility that Bdp-Chol exhibits periods of temporary confinement in generally simple-Brownian movements, by using two detection methods, developed by Simson et al. 63 and Sahl et al. 23. No significant confinements were found at the same time resolution of 0.5 milliseconds (Bdp-Chol in both intact and blebbed PMs and Cy3-DOPE in blebbed PMs) and at a time resolution of 33 milliseconds (Cy3-DOPE in intact PMs) (results not shown), further indicating that these molecules undergo effective simple-Brownian diffusion at these time resolutions. Therefore, the answer to the question of whether Bdp-Chol is trapped in nanoscale domains (the second aim of this work) is ‘no’ in the examined time scales longer than 0.5 milliseconds.

### **The HASM-cell PM** (1): ***peculiar diffusion of Bdp-Chol and Cy3-DMPE is undetectable even at a lower temperature (ensemble-averaged behaviors in time scales of 20 and 300 milliseconds, respectively)***

**Figure 5:**
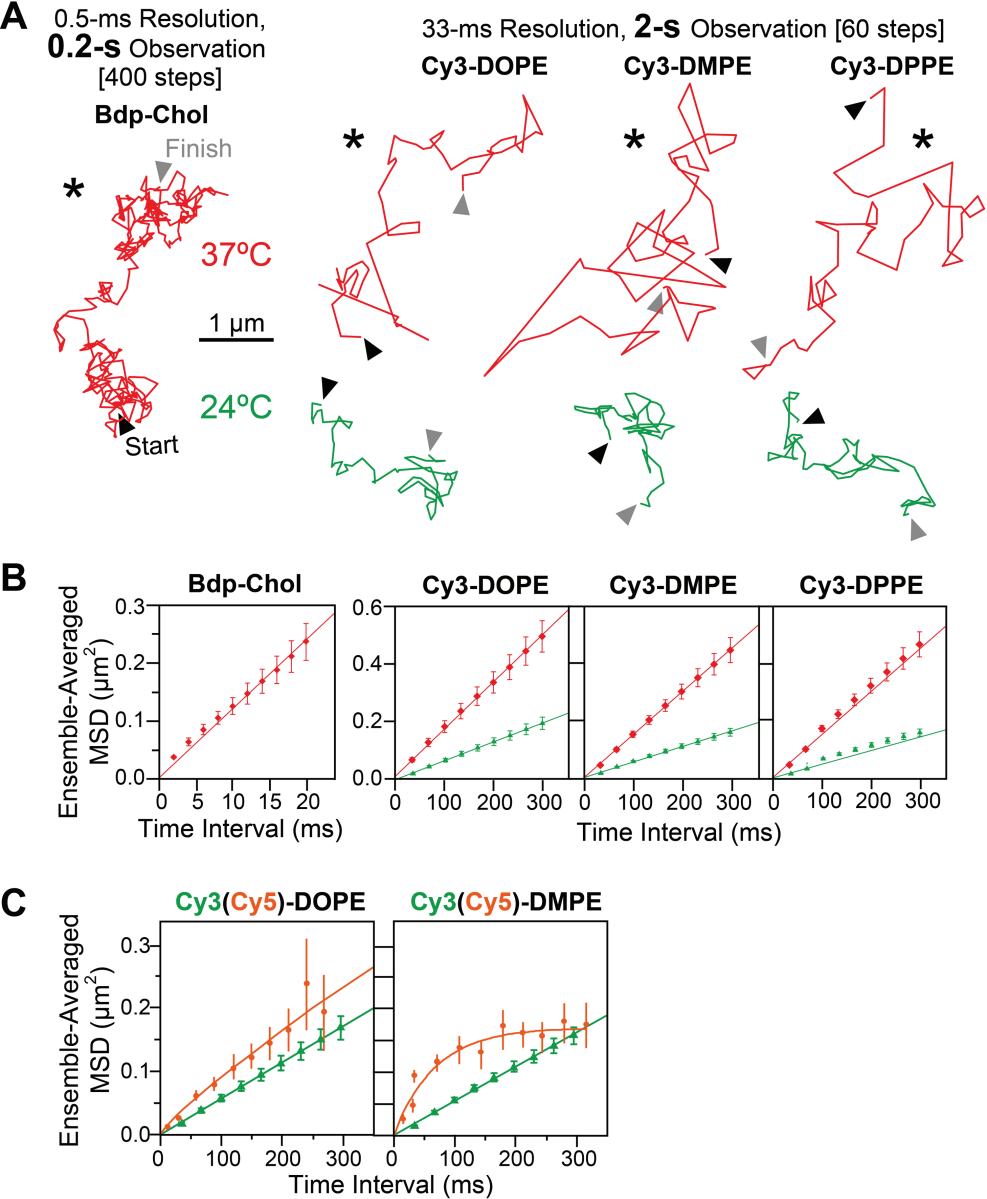
In the HASM-cell PM, Cy3-PEs, including Cy3-DMPE, and Bdp-Chol exhibited simple-Brownian diffusion: analysis by ensemble-averaged *MSD-Δt* plots for comparison with the results by Schütz et al. A) Typical trajectories of Bdp-Chol, Cy3-DOPE, Cy3-DMPE and Cy3-DOPE in the HASM-cell PM (Bdp-Chol: 0.5-millisecond resolution × 400 points, 0.2-second length; Cy3-PEs: 33-millisecond resolution × 60 points, 2-second length), recorded at 37°C (red) and 24°C (green). Arrowheads indicate the start (black) and finish (gray) points. B) The ensemble-averaged *MSD-Δt* plots for Bdp-Chol and Cy3-PEs at 37°C (red) and 24°C (green). Number of molecules examined (*n*): Bdp-Chol, *n* = 15; Cy3-DOPE at 37°C, *n* = 260; Cy3-DOPE at 24°C, *n* = 300; Cy3-DMPE at 37°C, *n* = 288; Cy3-DPE at 24°C, *n* = 318; Cy3-DPPE at 37°C, *n* = 589; Cy3-DPPE at 24°C, *n* = 255. Error bars represent standard errors. C) The comparison of the *ensemble-averaged MSD-Δt* plots obtained here (green) with those published in Schütz et al. (orange) for Cy3(Cy5)-DOPE and DMPE in this article (green). Error bars represent standard errors.

As described in *Introduction*, the HASM-cell PM may be unique, in that it might contain many more caveolae than the PMs of other cells 40,41, and thus contain 700-nm-sized raft domains or related domains that concentrate saturated phospholipids 36. In addition, it might not be compartmentalized by the actin-based membrane skeleton and its associated transmembrane proteins 36. Schütz et al. 36 reported that DMPE, which they assumed to be a raft-associated lipid, is confined within ∼700 nm domains in HASM cells, due to a raft-specific mechanism. Since this raft domain size is uniquely large, as compared with the sizes obtained by others, we suspected that the PM of the HASM cells may have a certain peculiarity, such as enhanced levels of raft domains (see *Introduction*; 40 41, 72). Therefore, we revisited this problem by observing Bdp-Chol and Cy3-PEs in the HASM-cell PM (typical trajectories shown in Figure 5A). Since Schütz's group performed their observations at room temperature, we observed Cy3-PEs at both 37°C and 24°C.

First, the ensemble-averaged behaviors of the probe molecules were examined, using the ensemble-averaged *MSD-Δt* plot (averaged over all examined molecules versus single-molecule *MSD-Δt*), following the approach by Schütz et al. We found that the ensemble-averaged *MSD-Δt* plots for Bdp-Chol and three Cy3-PEs, observed at both 37°C and 24°C, could be well-fitted by linear functions (p > 0.99, Figure 5B), indicating that they all undergo effective simple-Brownian diffusion in the 20- and 300-millisecond time scales (Figure 5B). These results are directly compared with those obtained by Schütz et al., who used Cy5-DOPE and DMPE, in Figure 5C (the use of Cy3 as compared to Cy5 should not affect the result; 73). The DOPE results were similar, but in our hands, DMPE also exhibited simple-Brownian diffusion, although Schütz et al. 36 reported confined diffusion of DMPE in the time scale of a few 100 milliseconds (an initial fast rise, which was much faster than that for DOPE, followed by the leveling off of the plot). This result was confirmed by Cy3-DPPE tracking data (Figure 5B, *extreme right*).

### **The HASM-cell PM (2): *peculiar diffusion of Bdp-Chol and Cy3-DMPE is undetectable even at a lower temperature (analysis by single-molecule MSD-Δt plots in the time scales of*** ≥***0.2 and*** ≥***1*** **second, *respectively)***

**Figure 6:**
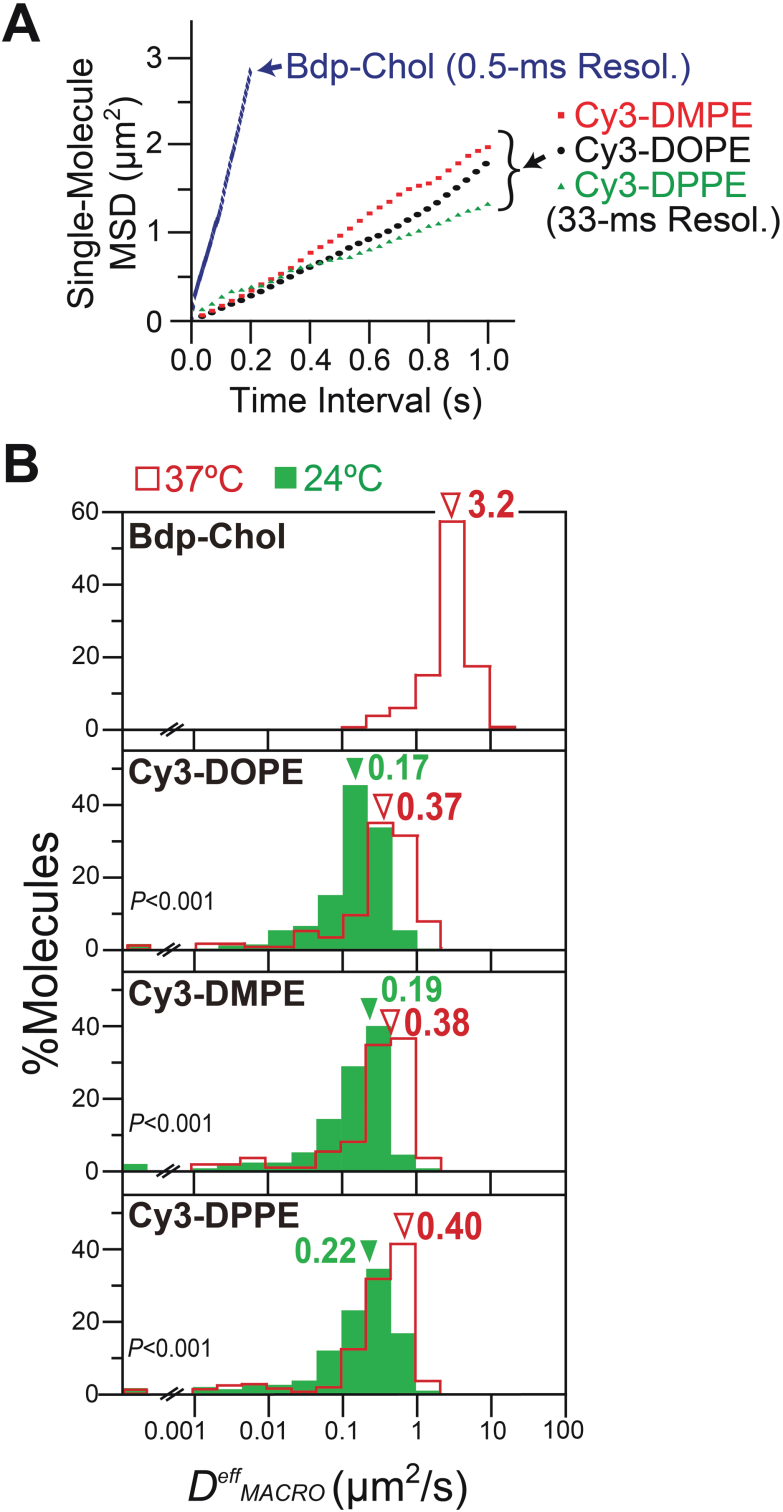
In the HASM-cell PM, virtually all Cy3-PEs and Bdp-Chol molecules, observed at 0.5- and 33-millisecond resolutions, exhibited simple-Brownian diffusion, whereas Bdp-Chol diffused ∼9× faster than Cy3-DOPE in the intact PM (comparison by median values): analysis by single-molecule *MSD-Δt* plots. A) Typical single-molecule *MSD-Δt* plots for Bdp-Chol and Cy3-DOPE in the intact PM of PtK2 cells. Each plot was obtained from the trajectory with an asterisk in Figure 5A, B) The distribution of *D^eff^_MACRO_* values for Bdp-Chol and Cy3-PEs (red open bars, 37°C; solid green bars, 24°C). Arrowheads indicate the median values. p: the results of the Mann–Whitney *U*-test. Number of molecules examined (*n*): Bdp-Chol in intact PM, *n* = 364; Cy3-DOPE at 37°C, *n* = 260; Cy3-DOPE at 24°C, *n* = 300; Cy3-DMPE at 37°C, *n* = 288; Cy3-DMPE at 24°C, *n* = 318; Cy3-DPPE at 37°C, *n* = 589; Cy3-DPPE at 24°C, *n* = 255.

Second, the *single-molecule MSD-Δt* plot (Figure 6A, rather than ensemble-averaged plots shown in Figure 5B,C) was used to classify each trajectory into one of the three diffusion modes (Table2), using the same method shown in Figure 2A. Practically all of the observed molecules in the HASM-cell PM exhibited simple-Brownian diffusion (rather than confined diffusion), including Cy3-DMPE observed at 24°C, in the time scales employed here (*nδt* ≥0.2 and ≥1 second for Bdp-Chol and Cy3-PEs, respectively; Table2), just like the Cy3-PEs in the PMs of other cell lines, including NRK, T24 and BHK cells. Namely, we did not detect any unique aspect of the HASM-cell PM, in terms of lipid diffusion. The third aim of this investigation was to clarify whether 700-nm raft domains where saturated phospholipids might be concentrated and confined exist in HASM cells (and in other cell types) as previously reported by Schütz et al. 36. Both the single-molecule analysis results obtained here and ensemble-averaged analysis described in the previous subsection indicate that there are no such half-micron-sized domains that confine saturated phospholipids neither in HASM cells nor in other cell types.

**Table 2:** Median values of *D^eff^_MACRO_* of Cy3-PEs (33-millisecond resolution) and Bdp-Chol (0.5-millisecond resolution), in the intact PM of the HASM cell, and the median compartment size evaluated by single-particle tracking of Gold-PEs (20-microsecond resolution). For the ranges of the observed values, see Tables S1 and 3, and Figures 6B and 7C. Median values are employed here for comparison with the mean values given in Tables S1 and 3 (*D^eff^_MACRO_*), and to account for the anomalous distributions of the compartment sizes shown in Figure 7C

Lipid	*D^eff^_MACRO_* (µm^2^/second) (SFMT; median)	Compartment size ([*LxLy*]^1/2^) (nm) (high-speed SPT; median)
DOPE	0.37	87
DMPE	0.38	86
DPPE	0.40	81
Mean of the median values^a^	0.39	85

Lipid	*D^eff^_MACRO_* (µm^2^/second) (SFMT; median)	Compartment size ([*LxLy*]^1/2^) (nm) (mean of PEs' median values)
Bdp-Chol	3.2	85

SFMT, single fluorescent-molecule tracking; SPT, single-particle tracking.

a Determined as the mean of the medians for three PEs.

This result implies that each Bdp-Chol and Cy3-PE trajectory in the HASM-cell PM can be characterized by a single effective diffusion coefficient, *D^eff^_MACRO_* (Figure 6B, Tables2 and 3). Bdp-Chol diffused at a *D^eff^_MACRO_* of 3.3 µm^2^/second, whereas the three Cy3-PEs diffused at a *D^eff^_MACRO_* of 0.45 µm^2^/second (median). Namely, the data shown in Figure 6B and Tables2 and 3 indicate that, in addition to the PtK2-cell PM, in the HASM-cell PM, Bdp-Chol diffused at much higher rates than Cy3-PEs (∼7× faster; *D^eff^_MACRO_* values of Cy3-PEs in the PMs of NRK, T24, BHK and COS-7 cells, in addition to PtK2 cells, are summarized in Table S1), and was only slowed by a factor of ∼2 from that in artificial membranes as well as that in blebbed PtK2-PMs [the diffusion coefficients of Bdp-Chol are 5.0–7.2 µm^2^/second in artificial liquid-crystalline membranes 15 and 6.3 µm^2^/second in the blebbed PtK2-cell PM; we were unable to produce the blebbed PM using HASM cells].

**Table 3:** Effects of partial actin depolymerization, actin stabilization and partial cholesterol depletion on the *D^eff^_MACRO_* values of Bdp-Chol and Cy3-PEs in the HASM-cell PM, observed at a time resolution of 0.5 and 33 milliseconds (in the time scales of ≥20 milliseconds and ≥1 second), respectively

Lipid	Temperature (°C)	Conditions	*D^eff^_MACRO_*^a^(µm^2^/second)	% Simple-Brownian^b^	*N*^c^
Bdp-Chol	37	Intact PM	3.3 ± 0.1	85	364
		Partial actin depolymerization	3.9 ± 0.3	79	81
		Actin stabilization	2.4 ± 0.2*^d^	74	92
		Partial cholesterol depletion	3.1 ± 0.3	66	79
Cy3-DOPE	24	Intact PM	0.19 ± 0.01	83	300
	37	Intact PM	0.44 ± 0.02	88	260
		Partial actin depolymerization	0.52 ± 0.03*	89	100
		Actin stabilization	0.27 ± 0.02*	71	132
		Partial cholesterol depletion	0.36 ± 0.02	88	113
Cy3-DMPE	24	Intact PM	0.21 ± 0.01	87	318
	37	Intact PM	0.45 ± 0.02	89	288
		Partial actin depolymerization	0.57 ± 0.04*	95	110
		Actin stabilization	0.31 ± 0.02*	78	96
		Partial cholesterol depletion	0.39 ± 0.03	94	112
Cy3-DPPE	24	Intact PM	0.26 ± 0.01	86	255
	37	Intact PM	0.47 ± 0.02	90	259
		Partial actin depolymerization	0.57 ± 0.04*	94	100
		Actin stabilization	0.28 ± 0.02*	72	92
		Partial cholesterol depletion	0.40 ± 0.03	95	110
Means for Cy3-PEs	24	Intact PM	0.22 ± 0.006	85	873
	37	Intact PM	0.45 ± 0.01	89	807
		Partial actin depolymerization	0.55 ± 0.006*	93	310
		Actin stabilization	0.33 ± 0.02*	74	320
		Partial cholesterol depletion	0.38 ± 0.02	92	335

a Mean ± SE.

b Fractions of trajectories classified into the simple-Brownian diffusion mode, determined in the time scale (*nδt*) of ≥1 second.

c Number of examined trajectories.

d Asterisks indicate statistically significant difference against the values found in the intact PM.

### The HASM-cell PM (3): PE *hop diffusion detected by ultrafast single Gold*-PE *tracking at a 20*-microsecond *resolution*

We considered the possibility that the Bdp-Chol diffusion in the HASM-cell PM is slowed due to the actin-induced PM partitioning, like that in the PtK2-cell PM. However, since actin-induced PM compartmentalization has not been established in the HASM-cell PM (rather, the prevalent presence of 700-nm domains has been suspected), we first examined it by ultrafast single *gold-particle* tracking of PEs, using a 40-nmø colloidal gold probe at a time resolution of 20 microseconds (50 kHz), with a single gold-particle localization accuracy of 19 nm 45,48. Nanogold-conjugated PEs (Gold-PEs) exhibited a *D^eff^_MACRO_* value that was only 1.3-fold smaller than that with Cy3-PEs (Figure S3), indicating the minimal detrimental effects of the gold probe (see *Materials and Methods)*.

**Figure 7:**
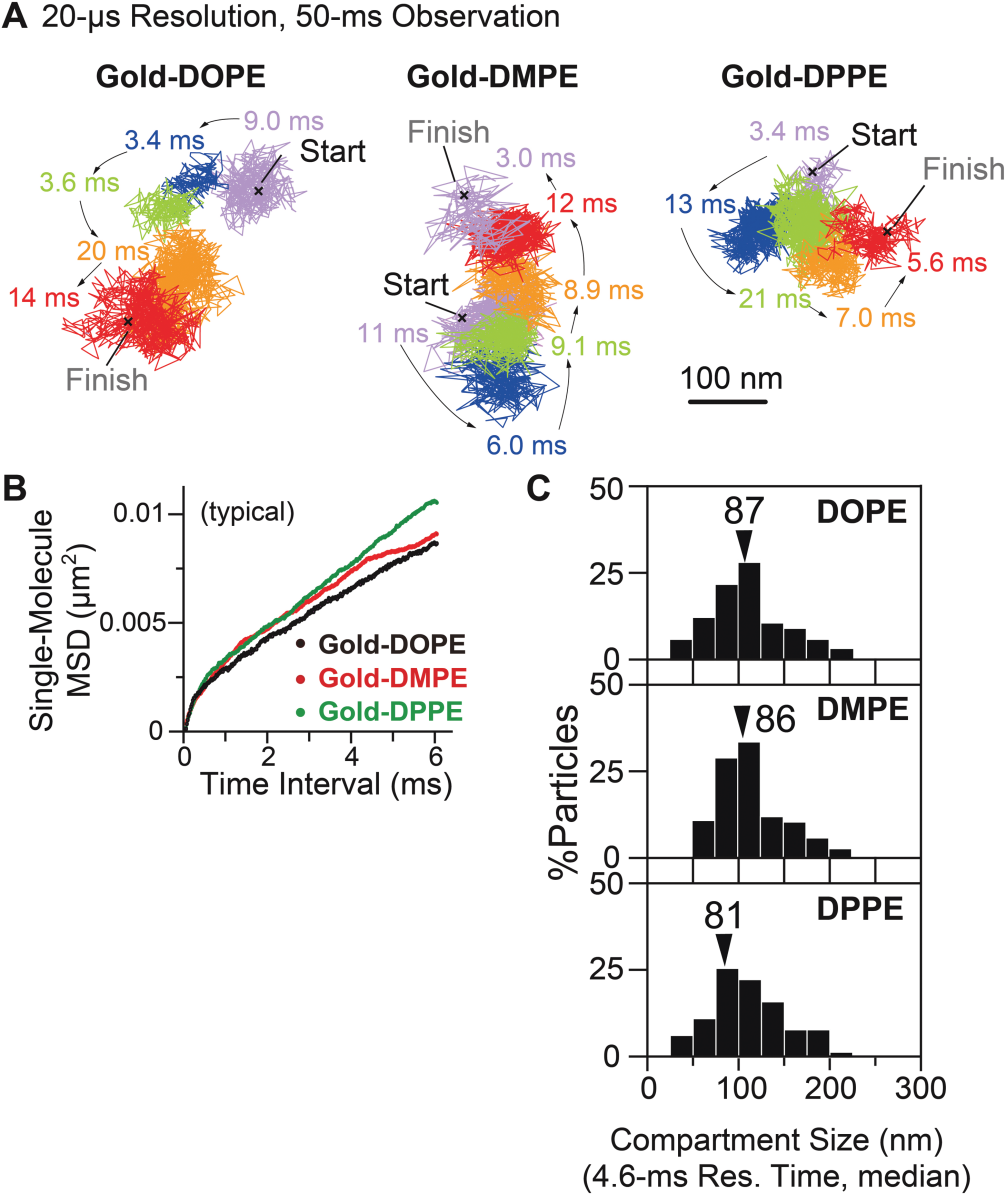
Ultrafast single-particle tracking of Gold-PEs observed at a time resolution of 20 microseconds revealed hop diffusion of these molecules in the HASM-cell PM. A) Typical trajectories of Gold-DOPE, DMPE and DPPE (2500 points in a 50-millisecond-long trajectory). Different colors indicate different plausible compartments detected by in-house software 45. The residency time within each compartment is shown. Scale bar, 100 nm. B) Representative single-molecule *MSD-Δt* plots for Gold-PEs on the time scale of 6 milliseconds (300 points), fitted by the theoretical curves for hop diffusion (hop-diffusion fitting). C) The distribution of the compartment size in the HASM-cell PM, obtained by hop-diffusion fitting. Arrowheads indicate median values.

Typical trajectories of Gold-PEs obtained at a 20-microsecond resolution are shown in Figure 7A. On the basis Based on their single-molecule *MSD-Δt* plots (Figure 7B), large majorities of the Gold-PE trajectories analyzed in the time scale of 1.5 milliseconds (n*δt* = 75 × 20 microseconds, Figure S4) were classified into the suppressed-diffusion mode, at variance with the results obtained in the time scale of ≥1 second (using Cy3-PEs). This is the reason why we employed the term ‘effective’ simple-Brownian diffusion in the previous subsections of this paper, to describe the diffusion observed at slower camera frame rates (i.e. at lower time resolutions).

Due to the classification result, each single-molecule *MSD-Δt* plot was fitted by the equation representing the model of idealized hop diffusion, in which a particle undergoes one-dimensional diffusion in the presence of equally spaced diffusion barriers of the same height (hop-diffusion fitting; 58). This hop-diffusion fitting provides the compartment size (the distance between barriers) *L*, the microscopic diffusion coefficient within a compartment (true diffusion coefficient in the absence of the compartments) *D*_micro_, and the long-term diffusion coefficient over many compartments *D*_MACRO_.

Using the hop-diffusion fitting, single-molecule *MSD-Δt* plots for Gold-PEs could be fitted well (Figure 7B), strongly suggesting that Gold-PEs undergo hop diffusion in the compartmentalized PM of the HASM cell, as in those of other mammalian cultured cells. The compartment size *L* was evaluated for each trajectory from the fitting, and its distribution for all of the trajectories is shown in Figure 7C. The average of the three median compartment sizes for the three Gold-PEs was 85 nm (Table2).

### The HASM-cell PM (4): *the actin-based membrane skeleton is responsible for the PE hop diffusion*

#### *The compartment size is affected by actin-modifying drugs, but not by cholesterol depletion: Ultrafast single Gold*-PE *tracking at a 20*-microsecond *resolution*

The involvement of actin filaments in the PE hop diffusion in the HASM-cell PM was examined. Again, the drug treatment conditions were finely tuned, and microscopic observations were performed during the period between 4 and 10 min after drug addition (0.1 µm latrunculin-B and 0.5 µm jasplakinolide) (see *Materials and Methods* for details).

**Figure 8:**
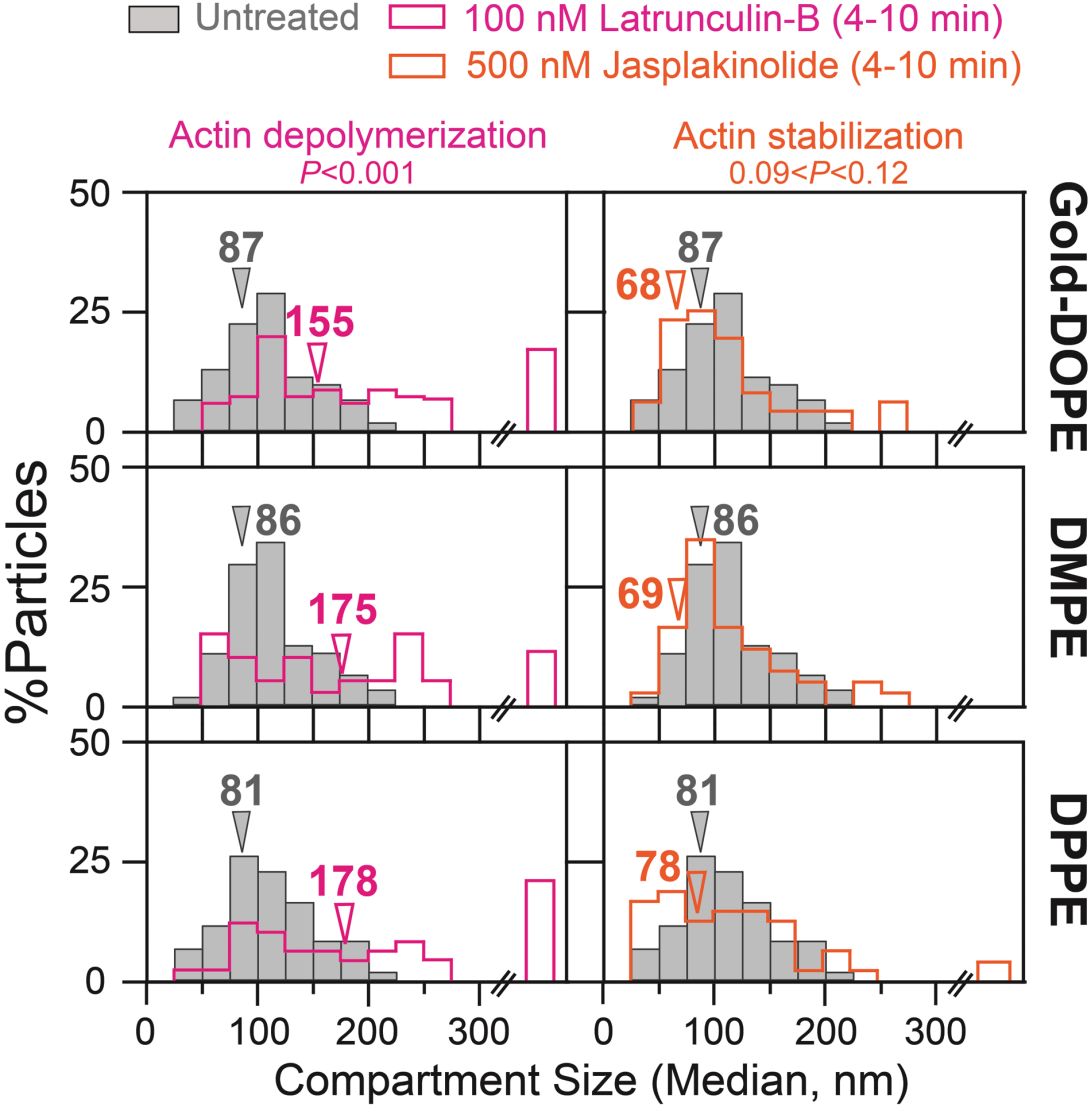
The effects of partial actin depolymerization and stabilization on the compartment size of the HASM-cell PM, evaluated by ultrafast single-particle tracking of Gold-PEs at a 20-microsecond resolution. Arrowheads indicate median values. Gray bars indicate the distributions before drug treatments (control). The results are summarized in Table4, including the numbers of examined particles. p: the results of the Mann–Whitney *U*-test.

The treatment with latrunculin-B induced ∼2-fold increases in the compartment size (evaluated by ultrafast single gold-particle tracking using Gold-PEs; Figure 8, *left* column, Table4). In contrast, as described in the previous subsection, it modestly increased the *D^eff^_MACRO_* values of Cy3-PEs by a factor of ∼1.2 (Table3).

**Table 4:** Effects of partial actin depolymerization, actin stabilization and partial cholesterol depletion on the compartment size in the HASM-cell PM, determined by Gold-PEs (median values), observed at a time resolution of 20 microseconds. For the spreads of the observed values, see Figures 8 and 9. Since the distribution of the compartment sizes after partial actin depolymerization is highly anomalous, median values, rather than mean values, are listed here. In all of the cases shown here, 78–95% of the trajectories were classified into the hop/confined diffusion mode in the time scale (*nδt*) of 1.5 milliseconds

Lipid	Condition	Compartment size (nm, median values)	*N*^a^
Gold-DOPE	Intact PM	87	81
	Partial actin depolymerization	155*^b^	112
	Actin stabilization	68	52
	Partial cholesterol depletion	68	60
Gold-DMPE	Intact PM	86	74
	Partial actin depolymerization	175*	48
	Actin stabilization	69	46
	Partial cholesterol depletion	77	79
Gold-DPPE	Intact PM	81	88
	Partial actin depolymerization	178*	46
	Actin stabilization	78	48
	Partial cholesterol depletion	69	32
Mean (± SE) of medians for Gold-PEs	Intact PM	85 ± 2	243
	Partial actin depolymerization	169 ± 7*	206
	Actin stabilization	72 ± 3	146
	Partial cholesterol depletion	71 ± 3	171

a Number of examined trajectories.

b Asterisks indicate statistically significant difference against the values found in the intact PM.

The treatment with jasplakinolide, which significantly decreased the *D^eff^_MACRO_* values of Cy3-PEs by ∼35% (Table3), did not affect the compartment size in a statistically significant manner (Figure 8, *right* column, Table4). Note that the compartment size determined from Gold-PE diffusion is rather insensitive to small variations in the microscopic diffusion coefficient of Gold-PE within a compartment 19,45.

These results unequivocally showed that actin filaments are involved in partitioning the HASM-cell PM into 85-nm compartments and temporarily confining phospholipids within these compartments, thereby slowing the macroscopic diffusion of PEs (directly detected with Cy3-PEs, as shown in the next subsection *Effects on* *D^eff^_MACRO_* *of Bdp-Chol in the* *PtK2 PM*), without any detectable 700-nm confining domains for saturated phospholipids.

**Figure 9:**
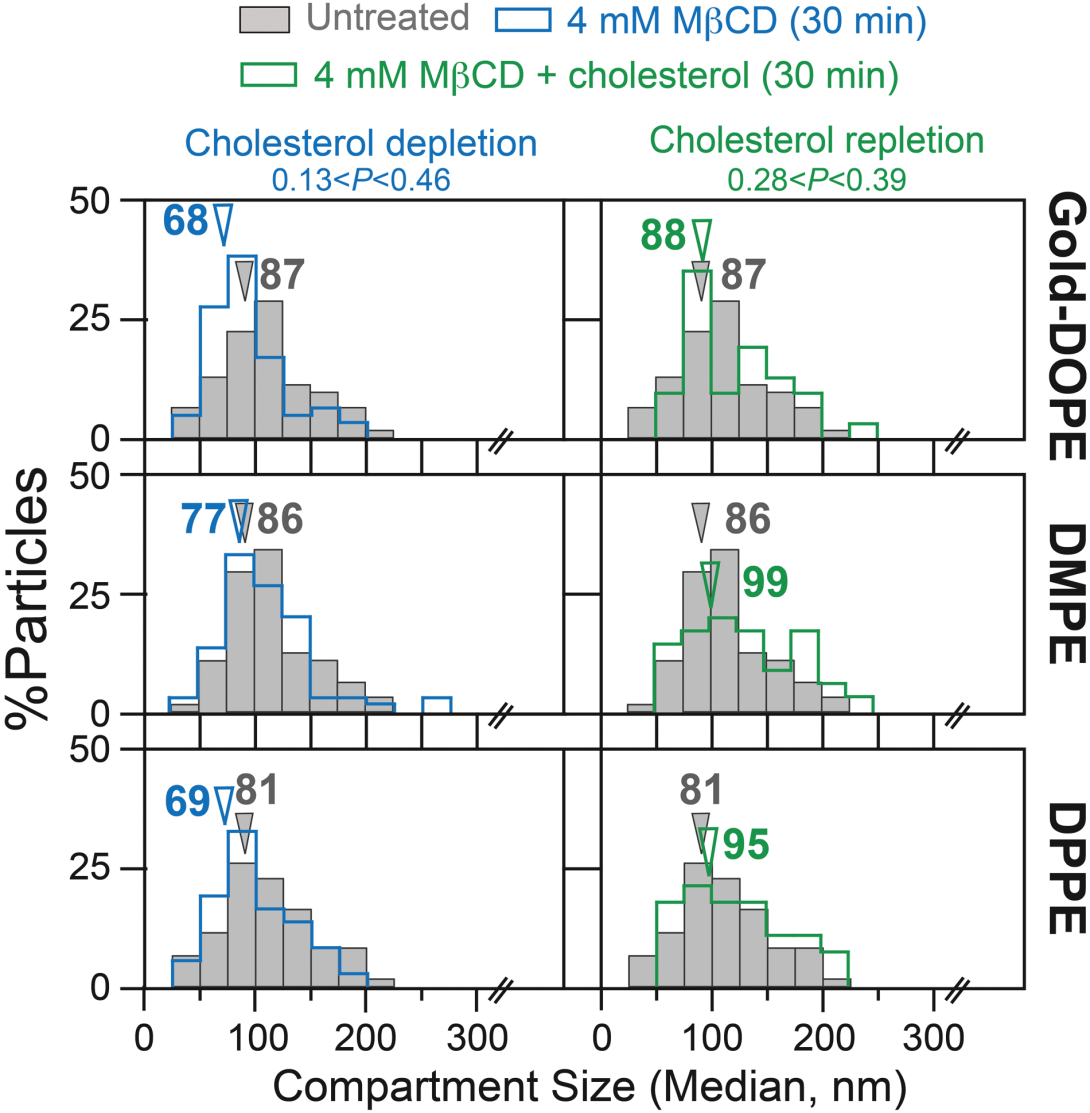
The effects of partial cholesterol depletion and subsequent cholesterol repletion after depletion on the compartment size of the HASM-cell PM, evaluated by ultrafast single-particle tracking of Gold-PEs at a 20-microsecond resolution. Arrowheads indicate median values. Gray bars indicate the distributions before drug treatments (control). The results are summarized in Table4, including the numbers of examined particles. p: the results of the Mann–Whitney *U*-test.

After partial cholesterol depletion with MβCD 68, as well as after the subsequent cholesterol replenishment, the compartment size evaluated by Gold-PEs was not affected at statistically significant levels (Figure 9, Mann–Whitney *U*-test). This is the first examination of the effect of partial cholesterol depletion on the PM compartment, and it was clearly not affected.

#### D^eff^_MACRO_ of Cy3-PEs is slightly but significantly affected by actin-modifying drugs: The actin-based membrane skeleton is responsible for the slowing of Cy3-PE diffusion in the intact PM from that in the blebbed PM or artificial lipid bilayer membranes

**Figure 10:**
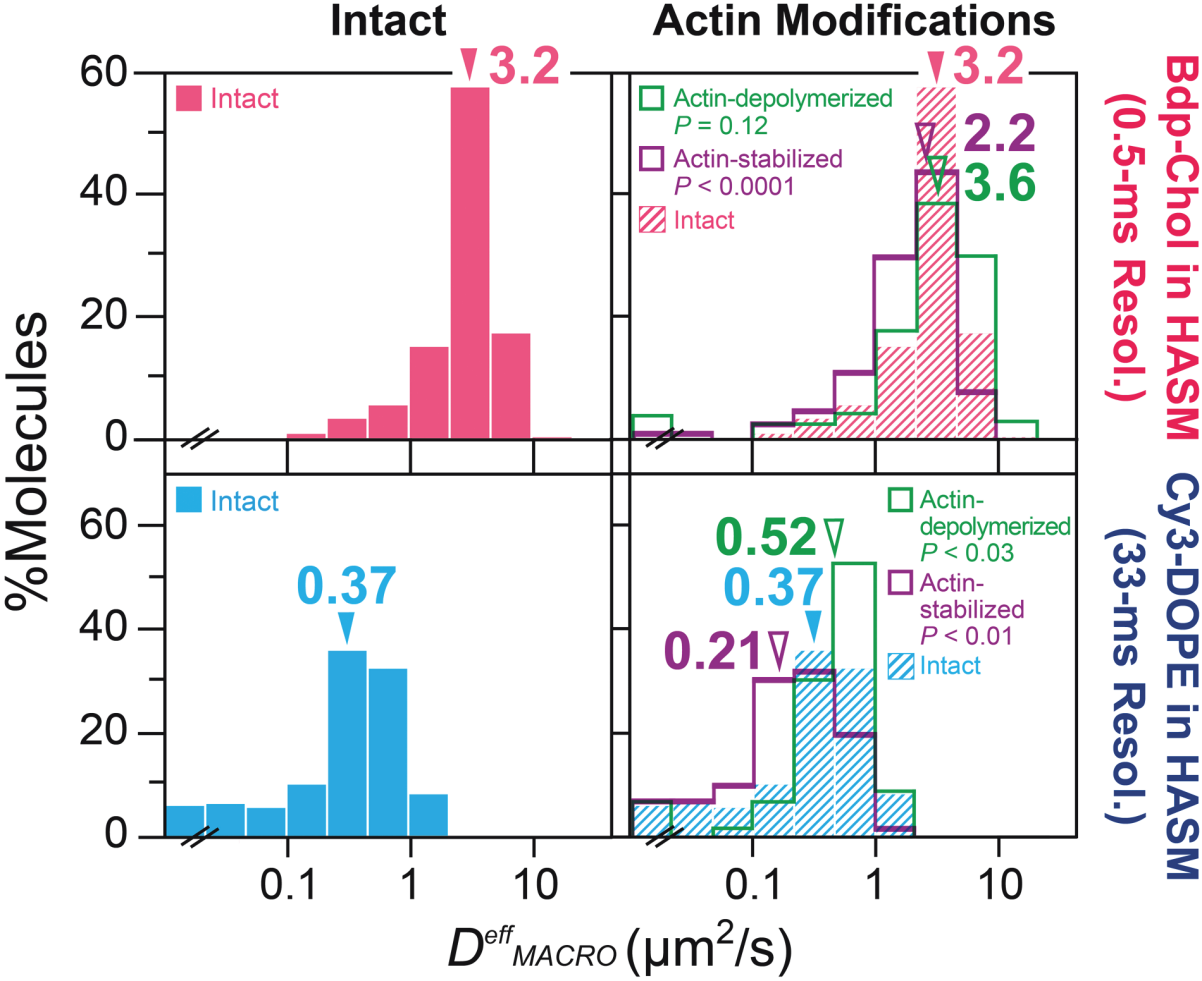
In the HASM-cell PM, Bdp-Chol diffused ∼9× faster than Cy3-DOPE in the intact PM (comparison by median values), probably due to the higher sensitivity of Cy3-DOPE diffusion to the actin-based membrane skeleton, as compared to that of Bdp-Chol diffusion. The distribution of the effective diffusion coefficient, *D^eff^_MACRO_*, for each trajectory of Bdp-Chol (top) or Cy3-DOPE (bottom) in the intact PM (both left and right), after partial actin-depolymerization (right), after actin stabilization (right) and in the blebbed PM (left) of HASM cells. Arrowheads indicate median values. p values indicate the Mann–Whitney *U*-test results against the results in the intact PM. See the summary in Tables2 and 3 (including the numbers of trajectories inspected).

After partial actin depolymerization by the latrunculin-B treatment, virtually all of the *Cy3*-PEs still exhibited simple-Brownian diffusion in the time scale of ≥1 second, with a ∼20% increase in *D^eff^_MACRO_* (Table3, the distribution for Cy3-DOPE is shown in Figure 10, *bottom right*), whereas actin stabilization by the jasplakinolide treatment led to a ∼35% decrease in *D^eff^_MACRO_* (the distribution for Cy3-DOPE is shown in Figure 10, *bottom right*) for all Cy3-PEs (*means for Cy3-PEs*, see Table3, which also includes the data obtained at 24°C). These results clearly indicate that the actin-based membrane skeleton, rather than the half-micron-sized domains that confine and concentrate saturated phospholipids proposed previously 36,37, is involved in slowing the phospholipid diffusion in the HASM-cell PM, strongly supporting that the actin-induced fence-picket model is applicable to the PM of the HASM cell.

This has now been firmly established by the use of six probes (DOPE, DMPE and DPPE conjugated to Cy3 or nanogold particles). Therefore, these results give a clear ‘yes’ answer to our initial question (iv-a) of whether the PM partitioning-compartmentalization by the actin-based membrane skeleton exists in the HASM-cell PM.

**Figure 11:**
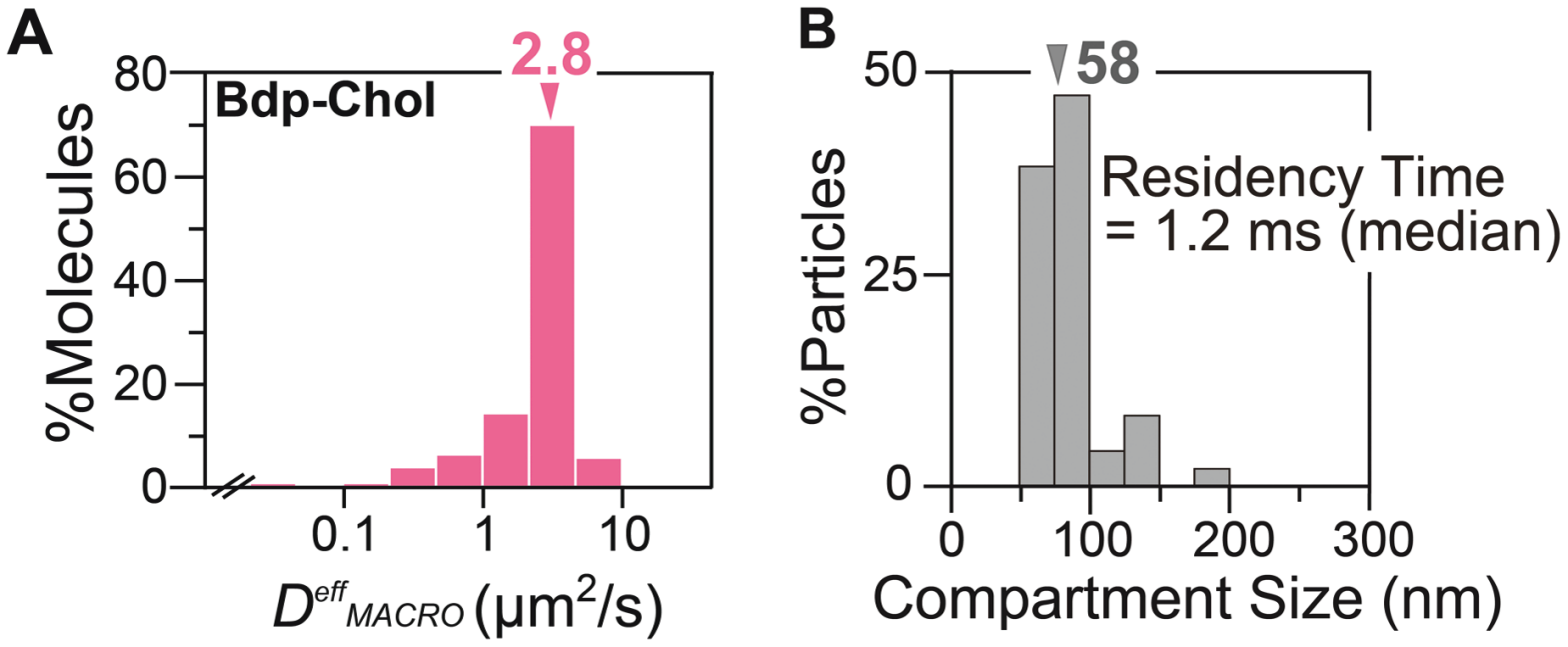
In the COS-7-cell PM, Bdp-Chol diffused as rapidly as in the PtK2- and HASM-cell PMs, and the COS-7-cell PM, like the PtK2- and HASM-cell PMs, is compartmentalized for phospholipid diffusion. A) The distribution of *D^eff^_MACRO_* values of Bdp-Chol in COS-7 intact PM (*n* = 164). The arrowhead indicates the median value. B) The distribution of the compartment sizes in the COS-7-cell PM, obtained by hop-diffusion fitting. The arrowhead indicates the median value.

The effects of actin-modifying drugs on *macroscopic* diffusion, even that of phospholipids and transmembrane proteins, have been found to be generally small 19,45, consistent with the data shown here (Figures 4 and 10). This is probably the reason why many scientists have previously been misled to conclude that the actin cytoskeleton is not involved in regulating the diffusion of membrane molecules, and that phospholipids undergo simple-Brownian diffusion, e.g. in PtK2-cell, HASM-cell and COS-7-cell PMs 12,23,36. However, using ultra high-speed single-molecule tracking with time resolutions better than 25 microseconds, hop diffusion of phospholipids has been clearly detected by statistical analyses (see Figures 7 and 11B for HASM- and COS-7-cell PMs, and Murase et al. 19 for the PtK2-cell PM), and the effects of actin-modifying drugs on the compartment size and the residency time within a compartment have also been clearly detected here, even for the HASM-cell PM [see Figure 8 and Table5; see Note superscript ‘a’ in Table5 for the equation to calculate residency time from the compartment size determined by ultrafast single Gold-PE tracking and the macroscopic diffusion coefficient of fluorescently labeled molecules. Table5 addresses our aims (iv-b) and (iv-c)].

**Table 5:** Summary of compartment sizes, *D^eff^_MACRO_* values and residency times of Cy3-PEs and Bdp-Chol in PtK2, COS-7 and HASM cells

Cell	Compt. size (Median, nm)	*D^eff^_MACRO_* (Mean ± SE, µm^2^/second)		Residency time^a^ (milliseconds, calculated)	
	Gold-PEs	Cy3-PEs	Bdp-Chol	Cy3-PEs	Bdp-Chol
PtK2	43	0.32 ± 0.02	3.3 ± 0.08	1.4	0.14
Intact	(Ref. 36)	(Figure 4, Table1)	(Figure 4, Table1)		
+ 5 µm Latrunculin	ND^b^	0.43 ± 0.03*^c^	3.9 ± 0.1*	ND	ND
+ 25 µm Jasplakinolide	ND	0.23 ± 0.02*	3.5 ± 0.2	ND	ND
Blebbed PM (+ actin depletion)	ND	6.4 ± 0.01*	6.3 ± 0.3*	ND	ND
		(Figure 4, Table1) (Cy3-DOPE)	(Figure 4, Table1)		
COS-7	58	0.37 ± 0.02	2.8 ± 0.01	2.3	0.30
	(Figure 1B)	(Table S1) (Cy3-DOPE)	(Figure 1A, Table1)	(Cy3-DOPE)	
HASM	85	0.45 ± 0.01	3.3 ± 0.1	4.0	0.55
Intact	(Figure 7C)	(Figure 6B, Table3)	(Figure 6B, Table3)		
+ 100 nm Latrunculin	169*	0.55 ± 0.006*	3.9 ± 0.3	13	1.8
+ 500 nm Jasplakinolide	72	0.33 ± 0.02*	2.4 ± 0.2*	3.9	0.54
	(Figure 8)	(Figure 10, Table3)	(Figure 10, Table3)		

a Calculated as (*compartment size*)^2^/(4 × *D^eff^_MACRO_*) using the values given in this table: the compartment size was obtained by ultrafast single-gold particle tracking of Gold-PEs, whereas *D^eff^_MACRO_* was determined by single fluorescent-molecule tracking of Cy3-PEs and Bdp-Chol at time resolutions of 33 and 0.5 milliseconds, respectively.

b ND = not determined.

c Asterisks indicate statistically significant difference against the values found in the intact PM.

### The HASM-cell PM (5): *the actin-based membrane skeleton is responsible for the slowing of Bdp-Chol diffusion in the intact PM from that in the blebbed PM or artificial lipid bilayer membranes*

The results obtained by Cy3-PEs and Gold-PEs suggest that the slight slowing of Bdp-Chol diffusion by a factor of ∼2 in the HASM-cell PM, as compared with the rates in artificial membranes and blebbed PMs, could be induced by the actin-induced fence-pickets. This was directly examined. In the case of Bdp-Chol, actin stabilization with jasplakinolide significantly reduced the *D^eff^_MACRO_* of Bdp-Chol by ∼30% in the HASM-cell PM (Table3 and Figure 10, *top*). The effect of partial actin depolymerization with latrunculin-B on the mean value of *D^eff^_MACRO_* was not statistically significant, but the tendency to increase *D^eff^_MACRO_* by latrunculin treatment was seen in the distribution of *D^eff^_MACRO_* (p = 0.12; Figure 10, *top right*). This latrunculin effect on Bdp-Chol diffusion detected in the HASM-cell PM was largely consistent with that found in the PtK2-PM (Figure 4, *top right*) (although, in the PtK2-cell PM, the *D^eff^_MACRO_* of Bdp-Chol was not significantly influenced by actin stabilization with jasplakinolide).

If we assume the picket-fence model for the Bdp-Chol diffusion in the PM, then the effect of actin-induced membrane compartmentalization on Bdp-Chol diffusion is the reduction of *D^eff^_MACRO_* by a factor of about two, as compared to that for Cy3-PEs by a factor of ∼20. Namely, the picket-fence effect on Bdp-Chol diffusion is weaker than that on phospholipid diffusion, by a factor of ∼10 in terms of the macroscopic diffusion coefficient or the residency time within a compartment (Table5). This intrinsically weaker effect of the actin membrane skeleton in the intact PMs on the Bdp-Chol diffusion is probably the reason why we see much less additional effects of actin-modifying drugs on the macroscopic diffusion of Bdp-Chol, using jasplakinolide (actin stabilization) in the PtK2-cell PM and latrunculin-B (partial actin depolymerization) in the HASM-cell PM.

Despite these subtle variations, since we detected statistically significant changes with one of the drugs for each cell line (latrunculin-B for the PtK2 cells and jasplakinolide for the HASM cells), and also because, first of all, the PM blebbing followed by further f-actin depletion increased the Bdp-Chol diffusion rate (in PtK2 cells; the blebbed PM could not be formed successfully using HASM cells), we propose that Bdp-Chol diffusion is 2× slowed from that in the blebbed PM and the artificial lipid bilayer membranes, due to PM partitioning by the actin-based membrane skeleton.

These results suggested that the model of the actin-based fence-picket is applicable to the HASM-cell PM, and that actin-based fence-pickets are responsible for suppressing Bdp-Chol diffusion in the HASM-cell PM.

In addition, the influences of partial cholesterol depletion, using MβCD, on the *D^eff^_MACRO_* values of Cy3-PEs and Bdp-Chol were inspected in the HASM-cell PM. In essence, no statistically significant effects could be observed (Table3). These results are apparently at variance with the observations made in the PtK2-cell PM, where partial cholesterol depletion, performed in exactly the same manner, reduced the *D^eff^_MACRO_* values of both Bdp-Chol and Cy3-DOPE (Table1). However, these results are both consistent with most of the previous results in the literature, in which partial cholesterol depletion generally did not increase the *D^eff^_MACRO_* values of transmembrane and GPI-anchored proteins as well as cytoplasmic lipid-anchored molecules 48,69–71. Overall, consistent with the observations made in PtK2 cells and those reported in previous publications, we conclude that cholesterol-enriched raft domains under physiological conditions will not reduce the macroscopic diffusion rates of either raft-associated (Bdp-Chol) or non-raft-associated (Cy3-DOPE) molecules in the PMs of HASM cells.

### The rapid diffusion of Bdp-Chol in the PM is probably a general feature of Bdp-Chol behavior in the PM

To examine the generality of the rapid macroscopic diffusion of Bdp-Chol in the intact PM, the diffusion of Bdp-Chol was further examined in COS-7 cells (in addition to PtK2 and HASM cells). Bdp-Chol in the COS-7 PM exhibited simple-Brownian diffusion with a *D^eff^_MACRO_* of 2.8 µm^2^/second (Figure 1A, Tables1 and 5), which is comparable to 3.3 µm^2^/second in both PtK2 and HASM cells [Figures 4 (top) and 6B (top); Tables1 and 5]. These results indicate that the rapid Bdp-Chol diffusion in the intact PM is a general feature of the Bdp-Chol, and possibly that of native cholesterol as well.

This result again suggests that the actin-induced fence-pickets slow the Bdp-Chol diffusion in the COS-7-cell PM. However, is the COS-7-cell PM compartmentalized for lipid diffusion? This question is important because Lenne et al., by using FCS, concluded that the COS-7-cell PM is compartmentalized for the diffusion of transmembrane proteins, but not for phospholipid diffusion 21. However, at variance with the conclusion by Lenne et al., our analyses clearly detected hop diffusion for Gold-DOPE, providing a median compartment size of 58 nm (Figure 1B). Although the reason for this difference is unclear, we suspect that the confocal observation area (∼120 nm, due to their improved method), which is much greater than the compartment size (58 nm) in the case of the COS-7-cell PM, compounded by the faster hop diffusion of phospholipids as compared with that of transmembrane proteins, might have contributed to the insensitivity of FCS to phospholipid hop diffusion in the COS-7-cell PM.

## Discussion

### Phospholipids generally undergo hop diffusion in partitioned PMs

Several previous reports using advanced FCS and single-molecule imaging at slow rates concluded that phospholipids undergo simple-Brownian diffusion, and that the PMs are not partitioned for phospholipid diffusion in HASM, COS-7 and PtK2 cells 21–23,40,74–76 as well as in other cell types 77–79. However, the results in the present research and Murase et al. 19, using ultrafast single-particle tracking of Gold-PEs at a time resolution of 20 or 25 microseconds (Figures 9, and 1B and Murase et al. 19), revealed that the intact PMs of HASM, COS-7 and PtK2 cells were compartmentalized, and even phospholipids, the most basic molecules to form the membrane, undergo hop diffusion in these PMs. The previous publications by other groups reporting simple-Brownian diffusion of phospholipids in the intact PMs would probably have been apparent simple-Brownian diffusion, due to limitations of the methods. Phospholipids would appear to undergo simple-Brownian diffusion when they are observed at insufficient spatial and temporal resolutions.

The results obtained here for the PMs of HASM, COS-7 and PtK2 cells, using unique ultrafast single-molecule tracking with time resolutions of 20 microseconds for gold probes and 0.5 milliseconds for fluorescent probes, are consistent with the hop diffusion of phospholipids (and transmembrane proteins) between the PM compartments induced by the actin-based membrane skeleton ‘fence’ and its associated transmembrane protein ‘pickets’, found in all other cell types we examined previously 7,46,80. Therefore, we propose that the PM partitioning by the picket-fence effects is actually a universal feature of the PM.

### Extremely rapid diffusion of Bdp-Chol in the intact PM, slowed only by a factor of two from that in the blebbed PM, due to actin-based fence-pickets

Bdp-Chol was found to undergo very rapid diffusion in the intact PM (2.8–3.3 µm^2^/second for PtK2, COS-7 and HASM cells), which was slowed only by a factor of ∼2 from that in the blebbed PM (6.3 µm^2^/second for PtK2 cells) or in artificial liquid-crystalline membrane bilayers (5.0–7.2 µm^2^/second; 15) (see Table5 for a summary of diffusion parameters). These values should be compared with those for Cy3-DOPE: 0.32–0.45 µm^2^/second for the intact PMs of PtK2, COS-7 and HASM cells, which are smaller than 6.4 µm^2^/second in the blebbed PM of PtK2 cells; i.e. a reduction by a factor as large as ∼20.

It should be clearly recognized that Bdp-Chol and Cy3-DOPE diffused at similar rates in the PtK2 blebbed PMs (6.3 and 6.4 µm^2^/second, respectively). Namely, the much larger diffusion coefficient of Bdp-Chol, as compared to that of Cy3-DOPE (∼10-fold) in the intact PM, is not due to the greater intrinsic diffusion coefficient of Bdp-Chol in the bilayer part of the membrane.

On the basis of the picket-fence model, we propose that the 2×-slowed diffusion of Bdp-Chol in the intact PM, as compared with that in the actin-depleted blebbed PM and in artificial lipid membranes, is due to the actin-based fence-picket effect, although the effect on Bdp-Chol diffusion is much smaller than that on phospholipid diffusion (∼20× slowing).

This conclusion is supported by the results examining the effects of latrunculin-B and jasplakinolide on Bdp-Chol diffusion, although the effects were often slight. The weaker effect of these drugs on Bdp-Chol than on PEs is probably due to the generally smaller confining effect of the actin-based fence-pickets on Bdp-Chol diffusion. On the basis of the results reported by Mondal et al. 11, we consider that Bdp-Chol is located in both the outer and inner leaflets of the PM. However, the distributions of the Bdp-Chol diffusion coefficients did not exhibit two clear components, suggesting that Bdp-Chol generally diffuses at similar rates in both layers.

In summary, we propose that the actin-based fence-picket slows the diffusion of Bdp-Chol in the intact PM. In the blebbed PM, Bdp-Chol diffuses at a rate similar to that of Cy3-PEs. The slowing effect of the actin-based fence-picket is much smaller on Bdp-Chol than on Cy3-PEs.

We initially anticipated that Bdp-Chol would diffuse more slowly than a non-raft unsaturated phospholipid, Cy3-DOPE, due to its probable association with raft domains (as illustrated by its association with the DRM). However, surprisingly, Bdp-Chol diffused faster than Cy3-DOPE (or any Cy3-PEs) by a factor of ∼10 in the intact PM. In addition, despite our search for its temporary entrapment or immobilization, we detected no such indication in time scales longer than 0.5 milliseconds.

### Extremely high hop probability of Bdp-Chol once it enters the compartment boundary zone

Assuming the hop diffusion of Bdp-Chol in the PM, partitioned by the actin-based membrane skeleton and its associated transmembrane protein pickets, the residency times of Bdp-Chol in PtK2, COS-7 and HASM cell compartments were determined (calculated using the equation in Note superscript ‘a’ of Table5). Residency times of 0.14, 0.30 and 0.55 milliseconds were obtained, respectively, and should be compared with the Cy3-PEs' residency times of 1.4, 2.3 and 4.0 milliseconds, respectively (summarized in Table5).

Meanwhile, in the actin-depleted blebbed PM, and thus within a compartment, Bdp-Chol diffused at a rate comparable to those of Cy3-PEs, colliding with the compartment boundaries as often as Cy3-PEs. Therefore, the shorter residency time (more frequent hops) of Bdp-Chol, as compared with that of Cy3-PE in each cell type, indicates that the hop probability of Bdp-Chol, once it enters the compartment boundary zone, is 7–10 times greater than that of Cy3-PEs.

### How does Bdp-Chol rapidly pass through the inter-picket space at the compartment boundaries?

We again assume the picket-fence model for the PM partitioning, and the hop diffusion of Bdp-Chol in the compartmentalized PM. When Bdp-Chol crosses the compartment boundary, it will have to pass through the space between two adjacent transmembrane protein pickets. In such a small intermural space between two pickets, consisting of only several lipid molecules sandwiched between the two immobile pickets, the lipid movement is expected to be greatly suppressed due to the hydrodynamic friction-like effect at the surface of the immobile picket proteins 81,82, and at the same time, the local lipid packing would be enhanced 83,84. Meanwhile, along the depth direction in the membrane (along the bilayer normal), the most crowded area is where the glycerol-backbone of the phospholipid is located 85. It follows then that, in the narrow inter-picket space in the compartment boundary, the friction from other lipids (very local viscosity) is largest in the glycerol-backbone zone (as compared with the friction near the membrane surface or in the middle of the bilayer). If Bdp-Chol is primarily located deeper in the membrane, and away from this glycerol-backbone area, as shown in the Bdp-Chol molecule located in the middle of the bilayer in Figure S2C, it can avoid the highest-friction z-area in the inter-picket space. Therefore, we suggest that such inner locations of Bdp-Chol in the PM allow its fast passage through the inter-picket spaces at the compartment boundaries.

Related to this proposition, Harroun et al. 86,87 performed a neutron scattering analysis of a bilayer composed of dipolyunsaturated 20:4–20:4 phosphatidylcholine and cholesterol, and found that cholesterol lies flat in the middle of the bilayer. Although this might not occur frequently in the PM, this result indicates the possibility that cholesterol can transiently lie flat in the middle of the bilayer. In this location, because Bdp-Chol avoids the most crowded z-zone (near the glycerol-backbone area) in the highly packed, viscous inter-picket area, Bdp-Chol would pass across the inter-picket space relatively quickly.

This raises the question of whether native cholesterol, in the intact PM, diffuses (hops across the compartment boundaries) as fast as Bdp-Chol. As described in *Results*, Khelashvili et al. 54 found that native cholesterol could move in toward the middle of the bilayer (along the bilayer normal, keeping the upright position) by ∼1 nm, which is as much as about 25% of the bilayer thickness. This result suggests that cholesterol can move out from the most crowded depth in the bilayer (glycerol-backbone region) by moving toward the center of the bilayer, and when this occurs, cholesterol might readily pass through the inter-picket space. We also expect that Cy3-PEs cannot sink into the membrane as much as cholesterol or Bdp-Chol, due to their greater hydrophilicity and the bulkiness of the phospholipid headgroup. If cholesterol often dynamically lies flat in the middle of the bilayer in the intact PM, as found in polyunsaturated lipid bilayers by Harroun et al. 86,87, then since it is away from the most crowded glycerol-backbone region in the membrane (located in the middle of the bilayer), it is plausible that native cholesterol can hop across the compartment boundaries (inter-picket space) quickly.

The diffusion of Bdp-Chol in the actin-depleted blebbed PM was not much different from that in artificial lipid membranes. The diffusion coefficients of Bdp-Chol in *artificial* liquid-crystalline membranes (5.0–7.2 µm^2^/second; 15) are comparable to those observed in the blebbed PM (6.3 µm^2^/second, the cholesterol mole fraction against total lipids in the PM is generally ∼40%; 88), as well as to those of non-labeled cholesterol in similar artificial membranes, as measured by ^1^H pulsed field-gradient magic-angle spinning NMR spectroscopy (3–7 µm^2^/second at 37°C; in this range, the diffusion coefficient is reduced with an increase of the cholesterol concentration in the membrane, in the range of 5–50 mol% cholesterol; 18). Therefore, it is likely that native cholesterol diffuses in the blebbed PM in a similar manner to Bdp-Chol in the blebbed PM.

### 85-nm actin-induced compartments, rather than ∼700-nm raft domains, exist in the HASM-cell PM

In the HASM-cell PM, where some peculiarities in lipid diffusion were expected from the literature, we found that three kinds of Gold-PEs underwent typical hop diffusion over actin-dependent compartments, as found in other cell types, even at 24°C (Figures 7 and 8). The compartment size was 85 nm, rather than ∼700 nm, and was not cholesterol-dependent (Figures 7 and 9). The mean residency time of Cy3-PEs within a compartment was 4.0 milliseconds (Tables2 and 5).

**Figure 12:**
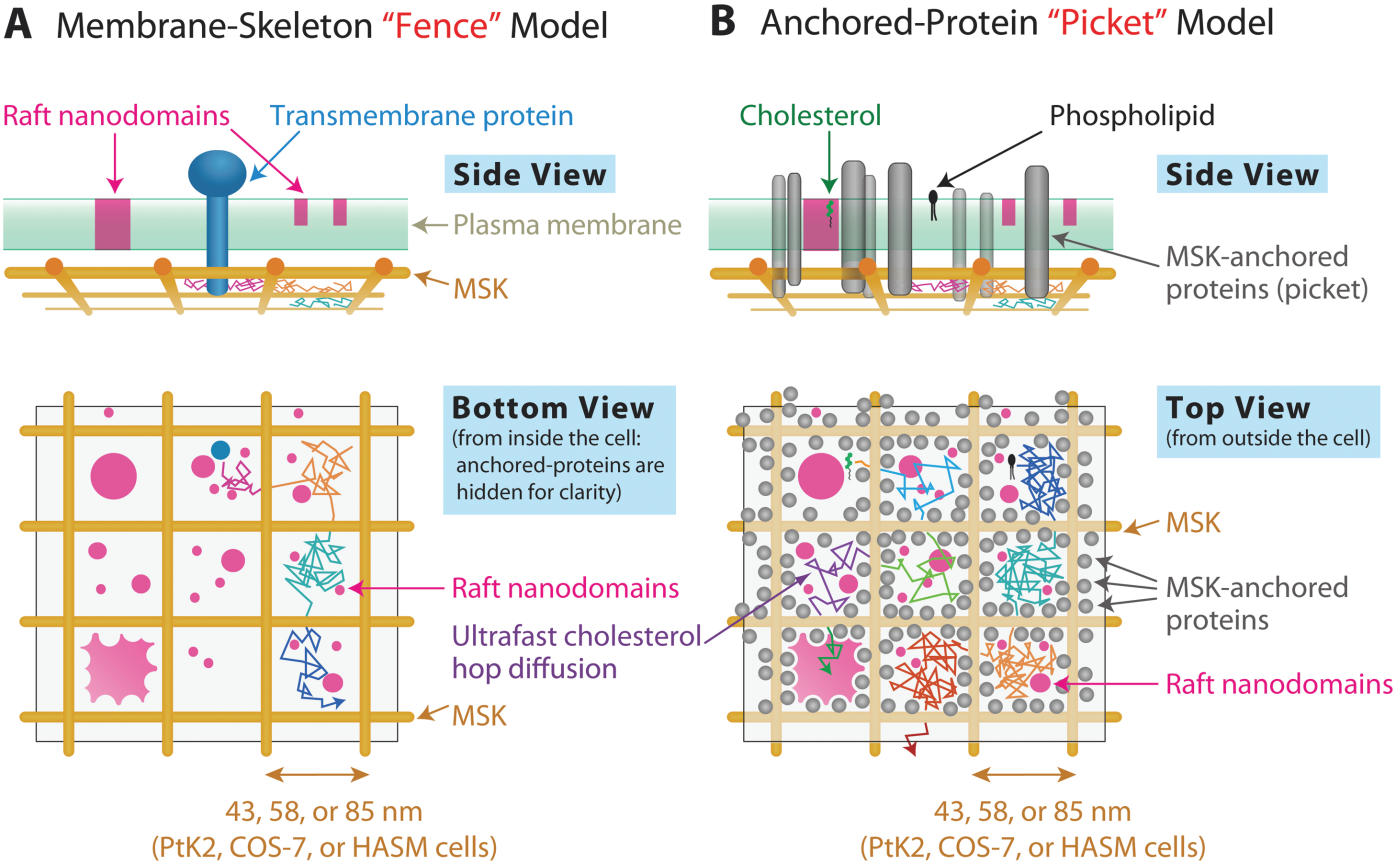
A schematic model for the coexistence of compartments generated by actin-based membrane skeleton and raft domains, which are contained in actin-induced compartments. According to this model, the PM is partitioned into domains (compartments) by the actin-based membrane skeleton (fences, A) and the transmembrane proteins anchored to the membrane skeleton (pickets, B), and raft domains coexist with the PM partitioning, being confined within the actin-induced compartments, whereas cholesterol only transiently associates with each raft, and readily hop across the compartment boundaries. Membrane molecules that collide with the membrane skeleton will be temporarily confined within a compartment, whereas even those that do not collide with the membrane skeleton will be temporarily confined within a compartment, due to the transmembrane picket proteins lining the membrane skeleton 47 by their steric hindrance and hydrodynamic friction effects. Previous results 25–28,101 together with those obtained here indicated that the sizes of the raft domains tend to be smaller than or comparable to those of the actin-induced compartments. This might be due to the blocking of the growth of raft domains by the non-raft-associated transmembrane domains of the picket proteins (many transmembrane proteins and raft domains are not likely to conform to each other; 6 7), which are aligned along the membrane skeleton actin filaments about 5–10 nm apart from each other. Due to the effect of these aligned picket proteins, each raft domain might be kept within a compartment, making the raft domains smaller than the compartment. As shown in (B) cholesterol is likely to hop over the compartment boundaries at a rate much higher than that of phospholipids. The compartment sizes for PtK2, COS-7 and HASM cells are 43, 58 and 85 nm, respectively.

The treatment of the HASM cells with 0.1 µm latrunculin-B for 4–10 min strongly affected the compartment size, increasing it from ∼85 to ∼170 nm (Figure 8), which is at apparent variance with the data from other groups 21,77,78. Although the cell treatment with 0.5 µm jasplakinolide for 4–10 min did not induce changes in the compartment size, consistent with our previous findings 45, it significantly slowed the *D^eff^_MACRO_*'s of Cy3-PEs. These results are inconsistent with both the crowding model 78 and the theory developed by Gambin et al. 89, but support the anchored-protein picket model (Figure 12).

Previously, it was proposed that raft-associated molecules are confined within cholesterol-dependent, <120-nm or <20-nm domains, with an overall time fraction of 50% or more (with a residency time for each confinement event of ∼20 or ∼2 milliseconds), in COS-7 and PtK2 cells at 37°C and 27°C, respectively 21–23. Meanwhile, Sezgin et al. 24 reported that such confinements do not involve raft domains, and Veljic et al. 70, Kenworthy et al. 69, Nishimura et al. 71 and Umemura et al. 48 reported similar diffusion coefficients for both raft- and non-raft-associated molecules. Our results using Bdp-Chol are more consistent with the latter results, because Bdp-Chol exhibited very fast diffusion, at a rate 8–10 times faster than those of the non-raft-associated Cy3-PEs in the intact PMs of both PtK2 and COS-7 cells (as in HASM cells). In addition, Bdp-Chol displayed no signs of confinement (entrapment within ∼120 nm domains for ∼20 milliseconds would readily be detectable by the present single fluorescent-molecule imaging, with a 0.5-millisecond resolution and a single-molecule localization accuracy of 19 nm). Clearly, further research is required.

### Coexistence of raft microdomains and membrane-skeleton-induced compartments

The PM compartments, delimited by the actin-based membrane skeleton and its associated transmembrane picket proteins, have been proposed to cover the entire cell membrane, except for the locations of other membrane structures, such as clathrin-coated pits, caveolae and cell-to-substrate and cell-to-cell adhesion structures 46,47. In the present work, such PM partitioning, even for phospholipids, was further confirmed in the PMs of PtK2, COS-7 and HASM cells, although other groups failed to detect phospholipid hop diffusion. The intercompartment contiguity is an important characteristic of the compartmentalized structure, as revealed by the close apposition of compartments in the trajectories of membrane molecules, observed at sufficiently high time resolutions (see Figure 7A). No significant diffusion-distance exists between adjacent compartments. In contrast, the contiguity of raft nanodomains with each other has generally not been seen or assumed 6,9,10,22,23,90,91.

The raft sizes in non-stimulated resting cells might range between several and several tens of nanometers in diameter (6 9, 10 21, 23; however, see 72), and are generally smaller than the sizes of the membrane-skeleton-dependent compartments (40–300 nm; 46). The present research revealed that the only possible exception, the case of the HASM cell, was not an exception at all. Since transmembrane proteins are generally raft breakers, the compartment boundaries formed by transmembrane picket proteins, lining the actin filaments, are likely to be non-raft regions. It follows then that the raft domains could not generally grow over the inter-compartmental boundary regions (Figure 2), consistent with the findings that the raft domains are usually smaller than the actin-dependent compartments, which are 85, 58 and 43 nm (medians) in the HASM, COS-7 and PtK2 cells, respectively, as determined in the present research and by Murase et al. 19. This concept is consistent with recent fluorescence microscopic observations, in which *raft-like, liquid-ordered-phase-like domains, with sizes much greater than the optical diffraction limit, were only visible in the blebbed PM, where actin filaments are largely depleted, at lower temperatures* 42–44,92,93. Namely, such micron-sized raft-like, liquid-ordered-phase-like domains could only be induced when the actin-based membrane skeleton is depleted, unequivocally showing that the membrane-skeleton-induced compartment boundaries regulate the growth of raft domains.

These results address our fifth objective of the present research, aiming to understanding the relationship between the cholesterol-enriched raft domains and the PM partitioning, induced by the actin-based membrane skeleton and its associated picket proteins (compartments). They coexist in the PM: the actin-induced PM partitioning exists throughout the PM and it confines individual raft domains within a compartment. Meanwhile, each individual raft-associated molecule will transiently enter and exit from the nanometer-scale dynamic rafts, and when it is dissociated from the raft domain, it can cross the actin-induced compartment boundaries and hop to an adjacent compartment. More research is needed to understand how exactly such molecular dynamics occurs and how the raft nanodomains and the actin-dependent compartments are organized in the PM and orchestrate the various PM functions.

## Materials and Methods

### Cell culture

PtK2 cells were grown in MEM (Sigma), supplemented with 0.1 mm non-essential amino acids, 1 mm sodium pyruvate (Gibco), and 10% FBS. HASM cells were grown in smooth muscle cell bottom medium (SmBM, Lonza), supplemented with 0.1% insulin, 0.2% hFGF, 0.1% hEGF, 0.1% gentamicin/amphotericin B and 5% FBS 94. COS-7 cells were grown in DMEM (Sigma), supplemented with high glucose (4500 mg/L) and 10% FBS. NRK and T24 cells were grown in Ham's F12 medium (Sigma) supplemented with 10% FBS. BHK cells were grown in MEM-α-medium (Gibco) supplemented with 10% FBS. Cells were plated on 12-mmø-glass-base dishes (for single fluorescent-molecule imaging) or 18 x 18-mm cover slips (for single-particle tracking), and were used 2 days later.

For the formation of blebbed PMs, PtK2 cells were washed twice with HBSS buffered with 2 mm PIPES at pH 7.4 (HP), and then incubated with 10 mm menadione (Sigma) dissolved in HP at 37°C for 5 h. Cytochalasin D (Sigma), dissolved in methanol, was then added at a final concentration of 10 µm, and the cells were incubated at 37°C for 1 h.

### Preparation and incorporation of Bdp-Chol, Bdp-DPPE and Cy3-PEs

Bdp-Chol was kindly provided by Dr. Robert Bittman, of the City University of New York. Bdp-Chol was first complexed with MβCD, at a molar ratio of 1:10 in HP, by vigorous mixing followed by sonication. The solution was then added to the cells in HP (final concentrations: 50 nm for the intact PM and 1 µm for the blebbed PM) set on the microscope stage. The cells were incubated with Bdp-Chol for 5 min at 37°C, and then washed twice with HP medium. Single fluorescent-molecule imaging of the incorporated probe molecules in the top PM was performed between 5 and 20 min after the addition of Bdp-Chol. As another method for incorporating Bdp-Chol into the PM, Bdp-Chol dissolved in methanol (347 µm) was mixed with HP by vigorous vortexing at a final concentration of ∼1 µm, and then added to the cells. No difference in the behavior of Bdp-Chol was found, regardless of the method employed.

Bdp-DPPE was purchased from Invitrogen, PEs were obtained from Avanti Polar Lipids, and the monovalent Cy3-probe was from GE Healthcare. Cy3 was covalently conjugated to the headgroup amine of the PEs, as described previously 19. Bdp-DPPE or Cy3-PEs dissolved in methanol (1 µm) were vigorously vortexed in HP (1–2 nm), and then added to the cells set on the microscope stage (37°C). The Bdp-DPPE or Cy3-PEs incorporated in the top PM were observed between 0 and 15 min after the addition of the probes. By 15 min after the probe addition, many fluorescently labeled endosomes became clearly visible, even near the top PM (in the bottom PM, they became visible immediately after the addition of Cy3-PEs, and many vesicles stayed near the PM, undergoing lateral diffusion and intermittent immobilization).

### Observations after cold Triton extractions of HASM and COS-7 cells

HASM and COS-7 cells were preincubated with fluorescent lipid probes [Cy3-PEs, Bdp(488)-Chol, or Bdpø-GM1 (in which the Bodipy-FL moiety is linked to a hydrophobic chain by an amide linkage; Life Technologies)], and were extracted with prechilled buffer (2.8°C) containing 1% (v/v) Triton X-100 (Biomedicals) on ice for 15 min (the temperature of the extraction medium was maintained at 2.8°C). After washing with PBS, the cells were observed by epifluorescence microscopy (IX70; Olympus), using a cooled CCD camera (Photometrics). For the fluorescence microscopic observation of endogenous GM1, after washing with PBS, the cells were incubated with 1 µm Cy3-conjugated cholera toxin B subunit in PBS for 4 min, and then were washed with PBS.

### Gold-probe preparation and cell surface labeling

The 40-nmø-colloidal-gold particles (BB International), conjugated with Fab fragments of anti-fluorescein antibodies (Molecular Probes), were prepared by mixing 50 μL of 13.2 µg/mL Fab in 2 mm phosphate buffer (pH 7.4) and 500 μL of colloidal-gold suspension (1.2 µg/mL Fab in the final mixture; 95). After incubating the mixture for 1 h at 26°C, the Fab-gold complex was further stabilized with 0.05% Carbowax 20M (Sigma). After two washes by centrifugation and resuspension in 0.05% Carbowax/2 mm phosphate buffer, pH 7.4, the conjugates were resuspended in 0.05% Carbowax/HP. The gold-probe suspension (∼0.02 nm of gold particles) was added to cells that had been preincubated with fluorescein-tagged DOPE, DMPE and DPPE for 3 min, prepared in the same manner as the Cy3-tagged phospholipids. Note that the fluorescein molecule was used as the ligand for the Fab antibody attached to the gold particle, rather than as a fluorescent tag.

The amount of Fab fragments mixed with the gold particles was optimized to minimize the crosslinking of fluorescein phospholipids by the Fab-gold probe, while maintaining reasonable specificity of Fab-gold binding to the cell surface. As the Fab concentration was reduced, the diffusion rate of the gold-DOPE complex determined at video rate increased, and it reached a plateau value of 0.37 µm^2^/second when 1.2 µg/mL of Fab was used. Therefore, to prepare the gold probes for the molecular diffusion observations, a 1.2 µg/mL final concentration of Fab was used.

These labeling conditions were optimal: in observations at the normal video rate, where both single fluorescent-molecule tracking and single-particle tracking can be performed with Cy3 and colloidal-gold probes, respectively, the *D^eff^_MACRO_* value determined at a 33-millisecond resolution with colloidal gold-conjugated PEs (Gold-PEs) was only 1.3-fold smaller than that with Cy3-PEs (Figure S3), indicating only small detrimental effects of the gold probe, which may include crosslinking the PE molecules, interacting with extracellular matrix proteins and/or extracellular domains of membrane proteins, and other general friction effects.

### Optical microscopy for single fluorescent-molecule tracking and single-particle tracking

All of the single fluorescent-molecule imaging and single-particle tracking analyses were performed for molecules in the top PM. Bdp-Chol and Cy3-PEs located in the top PM were observed at time resolutions of 0.5 and 33 milliseconds, respectively, using the home-built objective-lens-type TIRF microscope with an oblique-mode of illumination, based on an Olympus IX70 inverted microscope 19,45,48,57,96. The fluorescent images were projected onto the two-stage microchannel plate intensifier (C8600-03; Hamamatsu Photonics), coupled to a specially designed CMOS sensor-based camera (Photron) by way of an optical-fiber bundle. The observations were mainly performed only between time 0 and 15 min after the addition of the probes (0–15 min for PtK2 cells and 0–20 min for other cell lines).

The bottom PM was not observed, because it was difficult to differentiate the probes incorporated in the bottom PM from those bound to the coverslip (exhibiting temporary binding and sudden hops to locations nearby) and those internalized by endocytosis (these endosomes often remained near the bottom PM, exhibiting movements along the bottom PM with intermittent cessations of diffusion). The movements of these endosomes near the bottom PM were similar to those reported previously for some lipids in the PM of PtK2 cells 22,23. The endosomes formed near the top PM tended to disappear rapidly from the focal plane.

Gold-PEs bound to the top PM were observed by bright-field optical microscopy, and were recorded at a 20-microsecond resolution by a digital high-speed camera with a C-MOS sensor (FASTCAM-APX-RS, Photron), as described previously 19,45,48,57.

### Obtaining the trajectory from the single-molecule image sequence and the *MSD-Δt* plot for single molecules

The positions (*x* and *y* coordinates) of all of the observed fluorescent or gold probes were determined by a computer program that employs the method developed by Gelles et al. 97; the tracking of single molecules may end by probe detachment, photobleaching and temporary contrast decrease, due to fluctuations in the signal intensities of the probe and the background. For each trajectory, the MSD < *Δr*(*Δt_n_*)*^2^* > for every time interval was calculated according to the following formula 55,56,98:
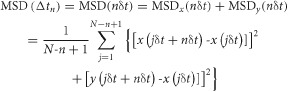


where *δt* is the frame time and *x*(*δt* + *nδt*), *y*(*jdt* + *ndt*) describe the particle position following a time interval, *Δt_n_* = *nδt*, after starting at position (*x*(*jδt*), *y*(*jδt*)), *N* is the total number of frames in the recording sequence, and *n* and *j* are positive integers (*n* determines the time increment). Namely, single-molecule MSD(*Δt_n_*) is defined in the following way. For a single-molecule trajectory consisting of *N* determined coordinates (*x*, *y*-positions) in a two-dimensional plane, all of the [*N-n* + *1*] partial trajectories of *n* consecutive positions (*n* ≤ *N*) were extracted. The MSD(*N*, *n*) was then calculated by averaging over the square displacements of *n* steps for all of these [*N-n + 1*] partial trajectories, and by varying *n*, the plot of MSD (*Δt_n_*) versus *nδt* (*δt* = the duration of each image frame) was obtained (Figure 2A; 55). When the overall length of the trajectory is important, the expression MSD(*N*, *n*) is employed for explicitly showing *N* in *MSD*(*Δt_n_*). MSD(0), the noise level for each trajectory, was estimated from the *y*-intercept of the straight line fitted for MSD(*2δt*), MSD(*3δt*) and MSD(*4δt*) and then was subtracted from each MSD 56.

### Classification of the diffusion mode, calculation of the diffusion coefficient, and analysis of the high-speed single-particle tracking trajectories

Comprehensive descriptions of the data analysis methods are available (see 45 48, 57). A statistical method for classifying each trajectory into suppressed diffusion, simple-Brownian diffusion, simple-Brownian diffusion with a drift, or immobile modes, based on the *MSD-Δt* plot, was described by Kusumi et al. 56. Briefly, all of the trajectories were first classified into mobile and immobile ones (usually less than a few % for lipid probes), and the mode-of-motion classification was performed only for the trajectories that were classified as mobile (see Figure 2, and its related text in the *Results* section).

Statistical classification of each individual trajectory was performed based on the parameter *RD*(*N*, *n*), which describes the long-term deviation of the actual single-molecule MSD(*N*, *n*) at time *nδt* from the expected MSD for simple-Brownian diffusion, based on the initial slope of the *MSD-Δt* plot, 4*D*_2-4_ *nδt*; i.e. *RD*(*N*, *n*) = MSD(*nδt*)/[4*D*_2-4_ *nδt*] (4*D*_2-4_ is the slope determined from a linear fit to the MSD values at the second, third and fourth steps of the elapsed time) (Figure 2A). See the *Results* section for details. In the case of molecules undergoing simple-Brownian diffusion, the average value of *RD*(*N*, *n*) is 1 (Figure 2A(1)). For molecules undergoing directed or suppressed diffusion, *RD*(*N*, *n*) tends to be greater or smaller than 1, respectively (Figure 2A, (2) and (3), respectively).

The *ensemble-averaged RD* values are ≪, ≈ and ≫1, when the molecules are undergoing suppressed diffusion, simple-Brownian diffusion and simple-Brownian diffusion with a drift (directed-diffusion mode), respectively. Figure 2A shows the theoretical curves for

1. simple-Brownian diffusion, MSD(*Δt*_*n*_) = 4*DΔt*_*n*_
2. directed-diffusion mode, in which a molecule moves in a direction at a constant drift velocity (*v_x_*, *v_y_*), with superimposed random diffusion, 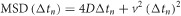


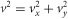
 and
3. confined diffusion (an extreme case of suppressed diffusion), in which a molecule undergoes Brownian diffusion while totally confined within a limited area (compartment; 0 ≤ *x* ≤ *L_x_*, 0 ≤ *y* ≤ *L_y_*) during the observation period. With an increase in *Δt*_*n*,_ the MSD(*Δt_n_*) plot levels off and asymptotically approaches a constant value, expressed as follows.

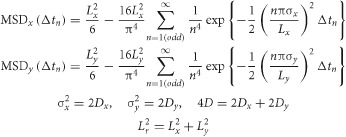


For the analysis of the trajectories obtained by using high-speed single-particle tracking with a 20-microsecond resolution and classified into the confined diffusion mode (under the analysis conditions employed therein), the *MSD-Δt* plots in the *x* or *y* directions were fitted with an in-house program based on the hop diffusion theory of Powles et al. 58, in which a particle undergoes diffusion in the presence of semipermeable barriers placed at equal distances (hop-diffusion fit; 19 45, 48 57).

The correct hop rate (or the residency time within a compartment), considering the effects of slight crosslinking by the Fab-gold probe, was evaluated from the macroscopic diffusion coefficient, determined by single fluorescent-molecule imaging with a fluorescent probe, and the compartment size, determined by single-particle tracking with a gold probe. Individual compartments for each trajectory were automatically identified by the computer program 45,48,57.

### Drug treatments

For partial cholesterol depletion 99, cells were incubated in HP containing 4 mm MβCD for 30 min at 37°C 45. Replenishment of cholesterol for cholesterol-depleted cells was performed by incubating the cholesterol-depleted cells in 10 mm MβCD-cholesterol complex (1:1) at 37°C for 30 min 60.

Determining the level of cholesterol depletion from the PM is difficult and, to the best of our knowledge, has never been accomplished. This is due to the difficulty of purifying the PM at the levels required for reproducibly quantitating cholesterol, and particularly for quantitatively comparing the cholesterol concentrations before and after the MβCD treatment. However, under the MβCD treatment conditions employed here, the cholesterol content in the whole cell was reduced by ∼35% in all of the cell types. This is consistent with our previous results 60,61, where we found that CD59 signaling was considerably suppressed. Since these cells do not contain detectable lipid droplets, our best guess is that cholesterol is depleted from the PM by ∼35% under the MβCD treatment conditions employed here.

For partial depolymerization or stabilization of actin filaments by drug treatments, the treatment conditions, including the drug selection, concentration and the treatment duration, were finely tuned, and single-molecule diffusion was carefully observed to detect the effects of the drugs on diffusion. The cells were incubated in HP (HP-CW for high-speed single-particle tracking) containing 0.1 µm (HASM cells) or 5 µm (PtK2 cells) latrunculin-B (Sigma) or 0.5 µm (HASM cells) or 25 µm (PtK2 cells) jasplakinolide (kind gift of G. Marriot, University of California, Berkeley), respectively, on the microscope 45. Microscopic observations were performed during the period between 4 and 15 min after drug addition, with the intention of conducting the observations only during the periods when the initial, small changes of the actin-skeleton are occurring. The proper (short) drug treatment duration is very important, because the initial perturbation of the actin membrane skeleton by the drug treatment tends to recover after 15 min 48,57.

The concentration ranges of the drugs, latrunculin-B and jasplakinolide, were selected so that their effects on the diffusion of *Cy3-PEs and Gold-PEs*, our control molecules, are detectable, without affecting the overall cellular morphology. Some readers might consider this to be an incorrect method, and that the drug effects on the actin-based membrane skeleton should be directly examined and the drug treatment conditions determined accordingly. However, as we clarified in our previous publications, to know the structure of the actin-skeleton associated with the PM cytoplasmic surface in *live cells*, the only available method was to observe the diffusion of transmembrane proteins and/or phospholipids at very high time resolutions (25 microseconds or better), to visualize the compartments, and thus clarify the compartment boundaries 7,45,57. Due to the abundance of actin filaments in the cytoplasm, there is no way to directly visualize the actin filaments on the PM cytoplasmic surface separately from the others in the bulk cytoplasm. For the actin filaments associated with the PM cytoplasmic surface *in fixed* (*dead*) *cells*, we developed an electron tomographic method in which three-dimensional structures of the PM and its associated actin filaments are reconstructed using a rapidly-frozen, deep-etched PM specimen, cleaved off from the cell body 7,100, but this is only applicable to the PM cleaved off from the cell body. However, importantly, when these two methods were applied to the same cell type, the distributions of the compartment sizes determined by diffusion data and those of the actin mesh sizes determined by electron tomography agreed well 7,100. This clearly indicates that observing the hop diffusion of transmembrane proteins and/or phospholipids at very high time resolutions is indeed a useful method when the parts of the actin-skeleton associated with the PM cytoplasmic surface in live cells must be observed, and this is exactly what we did here (the results shown in Figures 9 and 1B). Therefore, it is correct to observe the effects of actin-modifying drugs on Bdp-Chol diffusion under the conditions where their effects on Gold-PEs and Cy3-PEs are detectable.

As a result, the drug concentrations employed here were very different for PtK2 cells (5 and 25 µm for latrunculin-B and jasplakinolide, respectively) and HASM cells (0.1 and 0.5 µm, respectively), by a factor of 50. The effects of the actin-modulating drugs on *Bdp-Chol* diffusion were examined, using these concentrations.

Note that these treatment conditions with the actin-modifying drugs might not be applicable to the PMs of other cell types. Previously, in CHO cells, we found that a treatment with 10 µm cytochalasin D for 5–20 min hardly affected the hop diffusion, whereas a treatment with 1 µm latrunculin-A for 5–20 min considerably increased the compartment size 48. Meanwhile, the compartment sizes of the FRSK cell PM were sensitive to 13 µm cytochalasin D and 0.5 µm jasplakinolide for 5–15 min, but not to latrunculin-A at reasonable concentrations 19.

We think that the inability to detect any effects of actin-modulating drugs on phospholipid diffusion, as reported previously 21,77,78, was probably a consequence of neglecting the fine tuning of the experimental conditions. Schmidt and Nichols 77, Lenne et al. 21, and Frick et al. 78 all concluded that drug-induced actin modulation does not affect phospholipid diffusion, using *D^eff^_MACRO_* as a parameter. Previously, Fujiwara et al. 45 and Umemura et al. 48 reported the possibility that the failure of *D^eff^_MACRO_* to detect the effects of partial actin depolymerization (actin stabilization) by latrunculin (jasplakinolide) treatment is due to the combination of enlarged (reduced or similar) compartment size and increased (decreased) hop probability, which makes the *D^eff^_MACRO_* changes small and difficult to detect. Furthermore, the fact that the cells recover from the initial drug effects in 5–15 min was often missed, when low drug concentrations were used 48. At higher drug concentrations, the cells are frequently too debilitated to obtain meaningful measurements 48,57.

### Fluorescence quenching assay

CuTSP, purchased from Frontier Scientific, was dissolved in HP and the solution pH was adjusted to 7.0. HASM cells were labeled with Bdp-DPPE or Bdp-Chol, as described for normal single-molecule imaging. The fluorescence quenching assay was performed by replacing the normal HP medium for the HASM cell observations with the medium containing 3.3 mm CuTSP.

The total fluorescence intensity of Bdp in areas of 150 × 150 pixels was measured, and the background signal in the same area size, measured on the coverslip in the region near the cell, was subtracted, using the ImageJ software.

## Supporting Information

**Figure S1:** **Cold-Triton solubilities of Bdp-Chol and Cy3-PEs.** Epifluorescence microscopy images of HASM (A) and COS-7 (B) cells prelabeled with various Bdp-labeled lipids or Cy3-PEs, before (top) and after (bottom) cold Triton X-100 (1%) extraction. Endogenous GM1 was postlabeled with Cy3-conjugated cholera toxin subunit B, after Triton treatment (Cy3-CTXB). Scale bars, 40 µm. Among the examined molecules, only Bdp-Chol and native GM1 are insoluble with cold Triton. **Figure S2: Fluorescence quenching experiments of Bdp-Chol and Bdp-DPPE incorporated in the HASM-cell PM: design, results and interpretation**. A) Chemical structures of Bdp-Chol, Bdp-DPPE, and CuTSP and the model of Bdp-Chol and Bdp-DPPE incorporation in the plasma membrane. B) Typical single fluorescent-molecule images of Bdp-Chol (left) and Bdp-DPPE (right) incorporated in the HSM-cell PM before and after the addition of 3.3 mm CuTSP. The addition of 3.3 mm CuTSP instantaneously reduced the fluorescence intensity; Bdp-DPPE fluorescence totally disappeared; however, not to the zero level in the case of Bdp-DPPE, but to 34 (± 11)% of the original signal of Bdp-Chol (constant between 0 and 5 min after CuTSP addition). Scale bar, 5 µm. C) Schematic model for the locations of 1-palmitoyl-2-oleoyl-phosphatidylcholine, cholesterol and Bdp-Chol in the depth direction in the membrane. For the details of the Bdp-Chol location in the membrane, see the *Results* in the main text. **Figure S3: Examining the effect of gold probes on phospholipid diffusion, by comparing the *D^eff^_MACRO_*s of Gold-PEs (single-particle tracking) with those of the Cy3-PEs (single fluorescent-molecule tracking), in HASM cells**. The conditions for labeling the phospholipids with gold probes were optimized, by adjusting the concentration of fluorescein-phospholipids (here, fluorescein was not used as a probe for observing lipid motion, but was used as a hapten to conjugate the gold particle by way of an anti-fluorescein Fab) preincorporated in the plasma membrane and the amount of anti-fluorescein Fab fragments conjugated to the gold probes (see *Materials and Methods*; Refs 19, 45). Thus, the effect of crosslinking by the gold probes was minimized, while sufficient specificity for their binding to the cell surface was maintained. A) Typical trajectories that exhibited median values of *D^eff^_MACRO_* for each PE species. B) Histograms of *D^eff^_MACRO_* values for Cy3- (shaded bars) and *D_MACRO_* values for Gold- (open bars) PEs. C) Motional modes. A) The typical trajectories of the Gold- and Cy3-PEs in the plasma membrane of HASM cells, obtained at a 33-millisecond resolution. The diffusion coefficients *D_MACRO_* for the gold-tagged molecules were smaller than those for the Cy3-tagged molecules, by a factor of 1.3 on average (Figure S3B), with only slight decreases in the fraction of the simple-Brownian diffusion mode (Figure S3C). This result indicated that the gold probes induced only low levels of PE clustering. Since the reduction in *D^eff^_MACRO_* by the use of gold probes is limited, we concluded that high-speed single-particle tracking with gold probes, instead of single fluorescent-molecule tracking with Cy3 probes, can be employed for the diffusion studies of these phospholipids, on the time scale of less than 100 milliseconds (i.e. the time scale of the third spot in the *MSD*-Δ*t* plot at a 33-millisecond resolution). **Figure S4: The mode-of-motion classification of single Gold-PE trajectories obtained in the HASM-cell PM, showing that virtually all of the Gold-PEs (81–90%) undergo suppressed diffusion, which is most probably confined-hop diffusion**. The distributions of RD^20μs^(5000, 75)s for the Gold-PEs are entirely different from those for the Monte Carlo-generated simple-Brownian trajectories (top), showing that the majority of the Gold-PEs undergo suppressed diffusion in the time scale of 1.5 milliseconds. For more details, see the caption to Figure A. The numbers of trajectories examined = 81, 74 and 88 for Gold-DOPE, DMPE and DPPE, respectively. **Table S1**: *D^eff^_MACRO_* for Cy3-PEs in six cell lines, observed at a 33-millisecond resolution, complementing the results shown in Figures 4, 6B, 10 and 11A
