# Supplementary figures and images for: Ultrafast Diffusion of a Fluorescent Cholesterol Analog in Compartmentalized Plasma Membranes
Source: Traffic. 2014 Mar 11;15(6):583–612. doi: 10.1111/tra.12163 (PMC4265843; doi:10.1111/tra.12163)

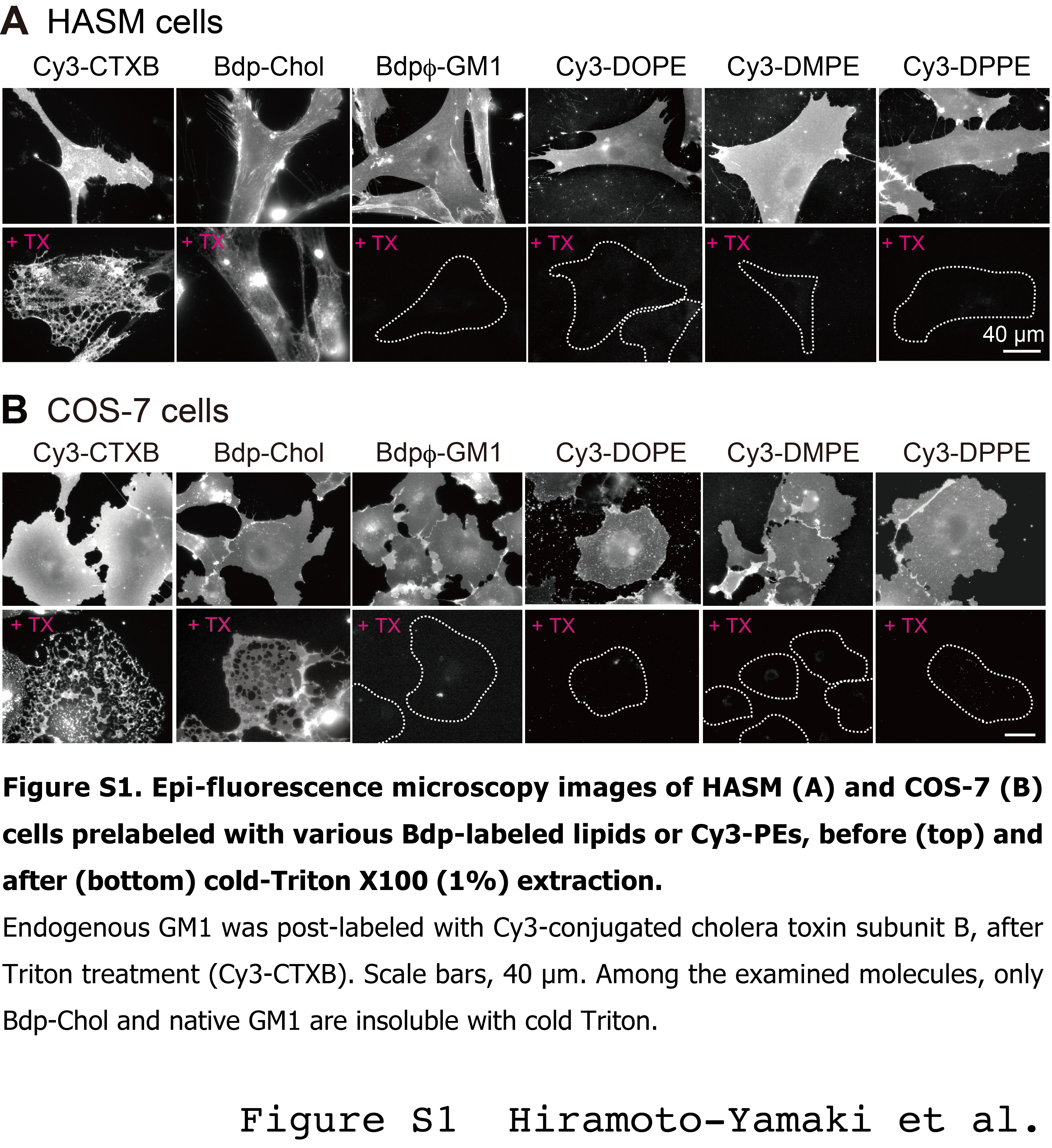

Supplement: Supplementary file 1 [file tra0015-0583-SD1.doc]

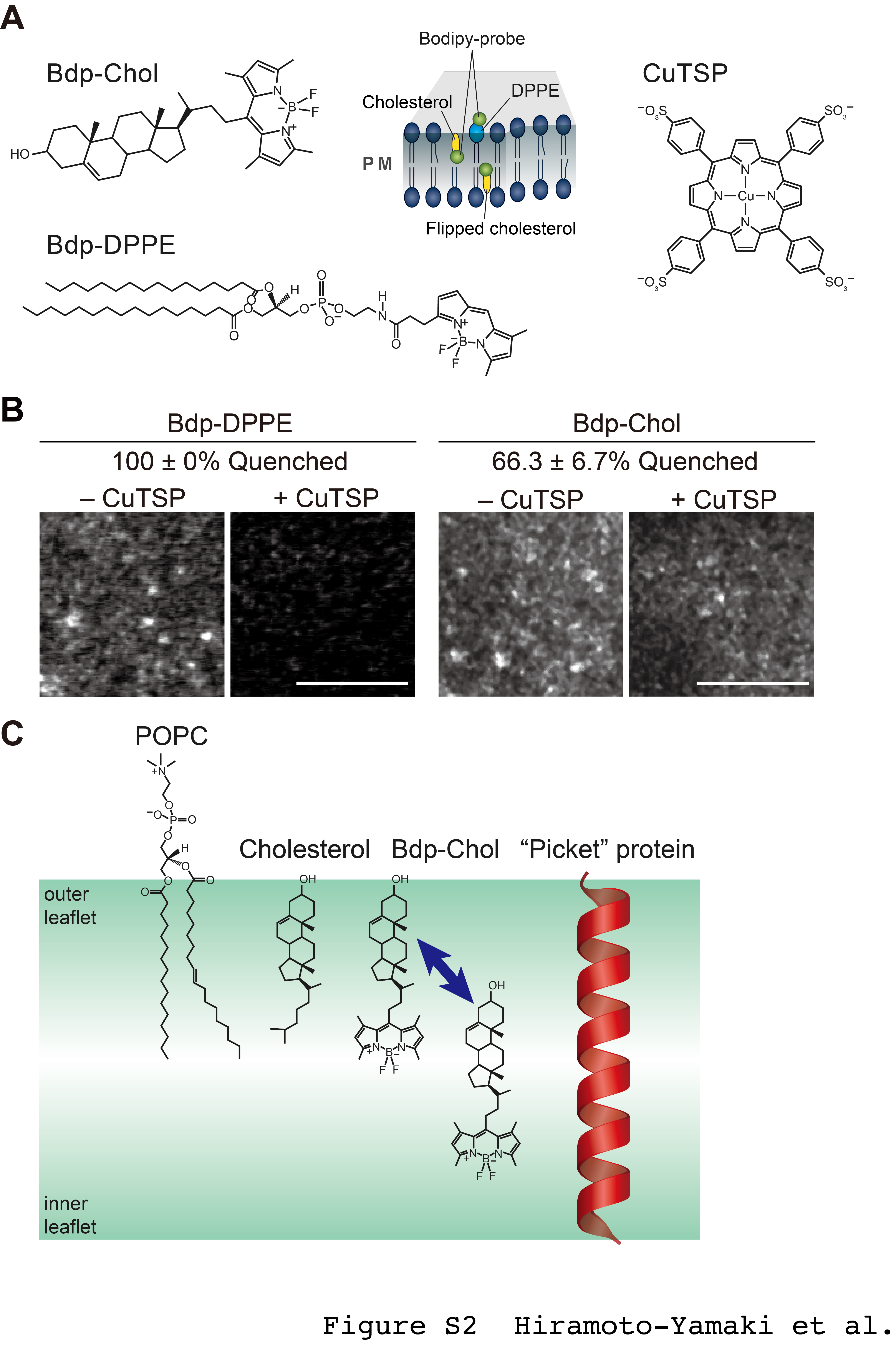


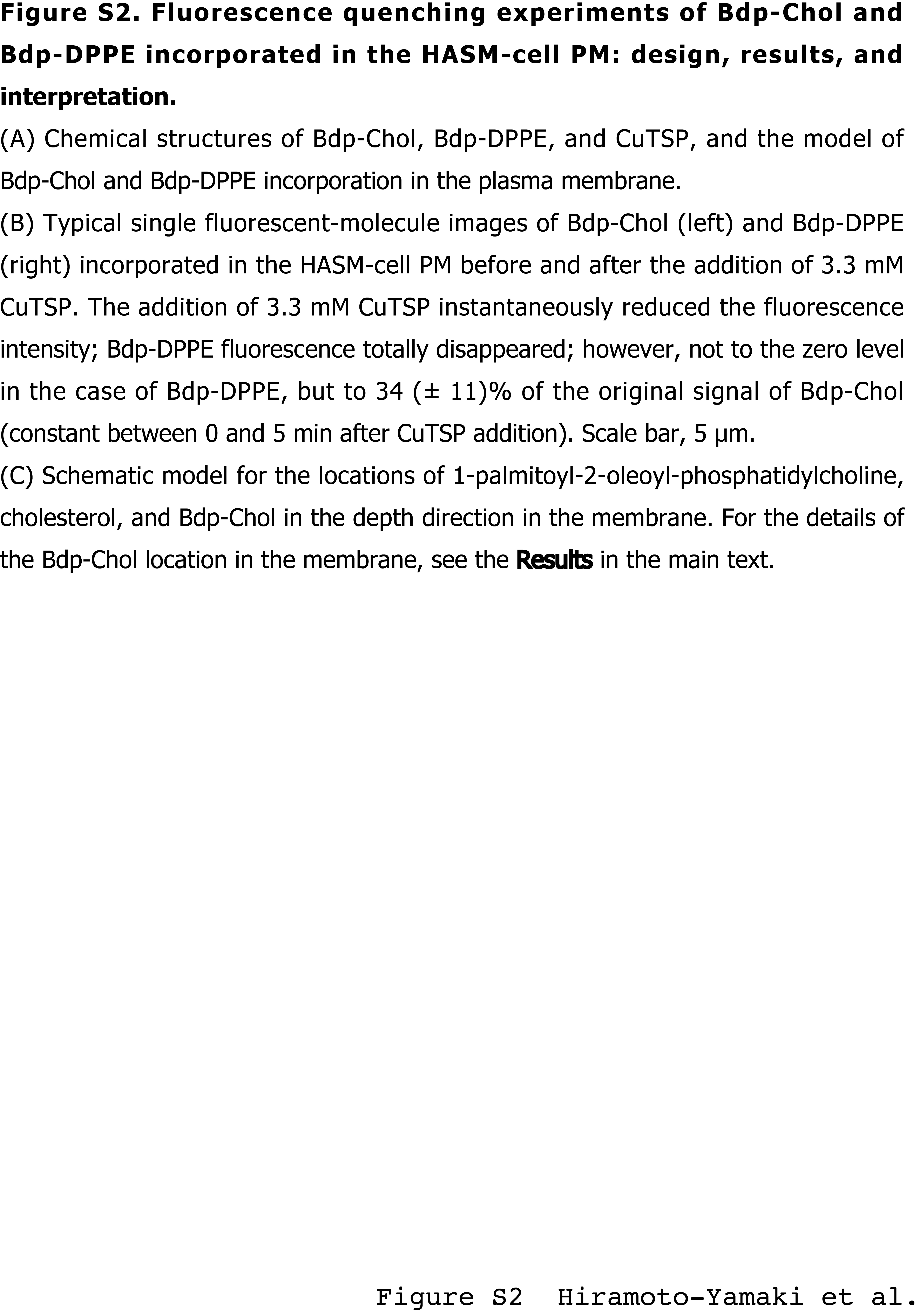

Supplement: Supplementary file 2 [file tra0015-0583-SD2.doc]

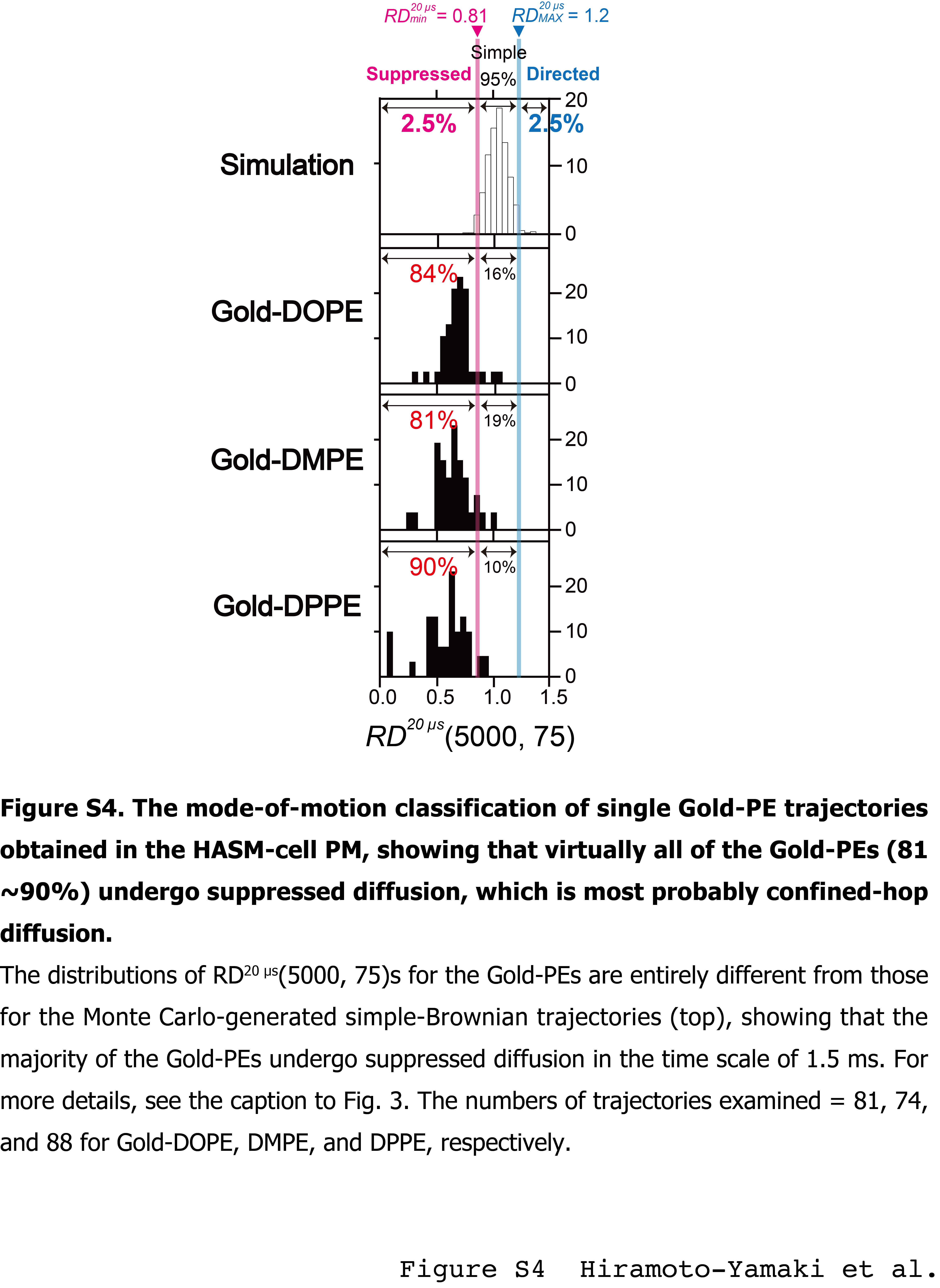

Supplement: Supplementary file 4 [file tra0015-0583-SD4.doc]
