# Supplementary material for: Ultrafast Diffusion of a Fluorescent Cholesterol Analog in Compartmentalized Plasma Membranes
Source: Traffic. 2014 Mar 11;15(6):583–612. doi: 10.1111/tra.12163 (PMC4265843; doi:10.1111/tra.12163)
Supplement: Supplementary file 3 [file tra0015-0583-SD3.doc]

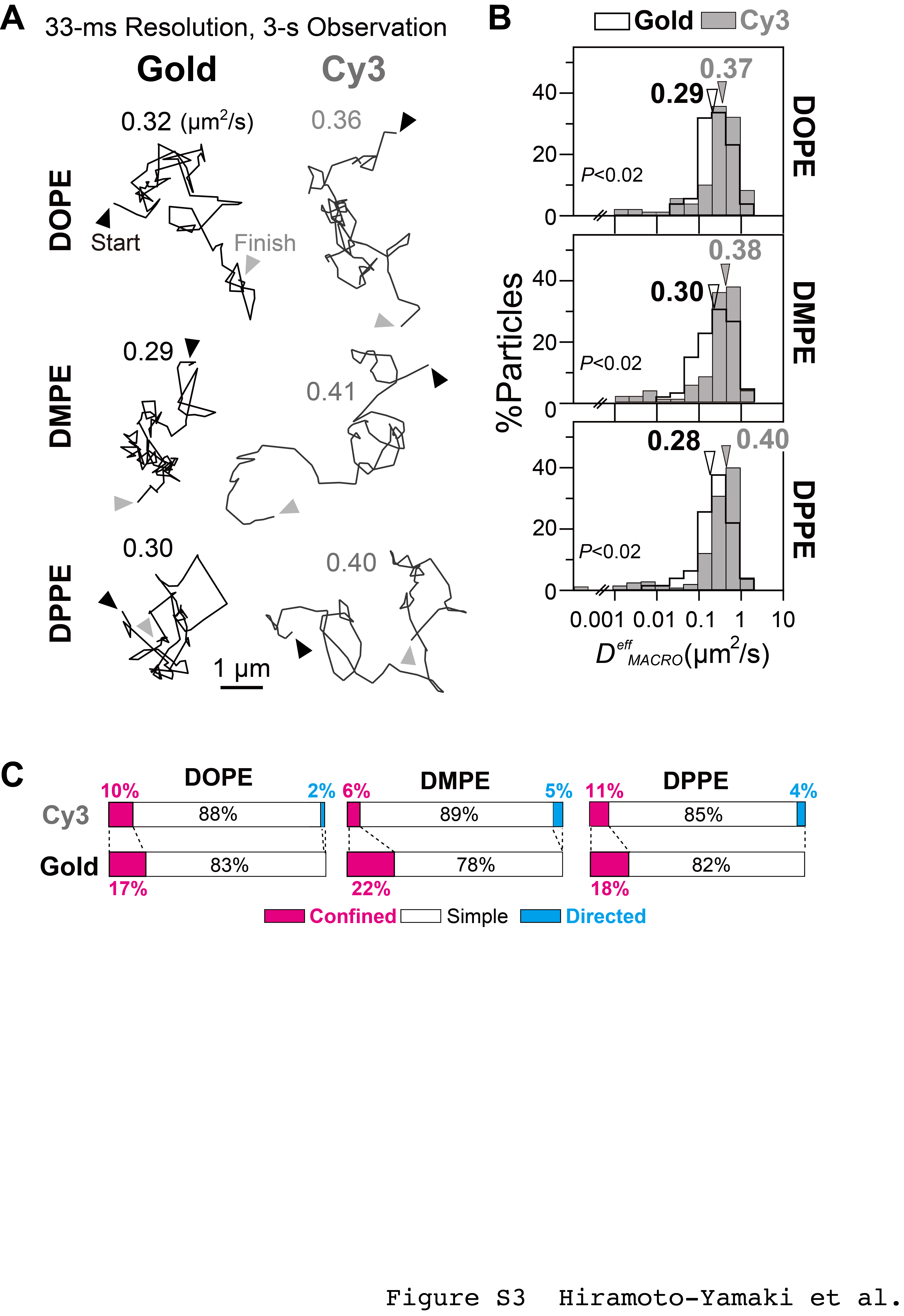


**Figure S3. Examining the effect of gold probes on phospholipid diffusion, by comparing the *D^eff^_MACRO_*s of Gold-PEs (single-particle tracking) with those of the Cy3-PEs (single fluorescent-molecule tracking), in HASM cells.**

The conditions for labeling the phospholipids with gold probes were optimized, by adjusting the concentration of fluorescein-phospholipids (here, fluorescein was not used as a probe for observing lipid motion, but was used as a hapten to conjugate the gold particle by way of an anti-fluorescein Fab) pre-incorporated in the plasma membrane and the amount of anti-fluorescein Fab fragments conjugated to the gold probes (see **Materials and Methods**; 19,45). Thus, the effect of crosslinking by the gold probes was minimized, while sufficient specificity for their binding to the cell surface was maintained.

**(A)** Typical trajectories that exhibited median values of *D^eff^_MACRO_* for each PE species.

**(B)** Histograms of *D^eff^_MACRO_* values for Cy3- (shaded bars) and *D_MACRO_* values for Gold- (open bars) PEs.

**(C)** Motional modes.

Fig. S3A shows the typical trajectories of the Gold- and Cy3-PEs in the plasma membrane of HASM cells, obtained at a 33-ms resolution. The diffusion coefficients *D_MACRO_* for the gold-tagged molecules were smaller than those for the Cy3-tagged molecules, by a factor of 1.3 on average (Fig. S3B), with only slight decreases in the fraction of the simple-Brownian diffusion mode (Fig. S3C). This result indicated that the gold probes induced only low levels of PE clustering. Since the reduction in *D^eff^_MACRO_* by the use of gold probes is limited, we concluded that high-speed single-particle tracking with gold probes, instead of single fluorescent-molecule tracking with Cy3 probes, can be employed for the diffusion studies of these phospholipids, on the time scale of less than 100 ms (i.e., the time scale of the third spot in the *MSD-∆t* plot at a 33-ms resolution).
