# Supplementary material for: Ultrafast Diffusion of a Fluorescent Cholesterol Analog in Compartmentalized Plasma Membranes
Source: Traffic. 2014 Mar 11;15(6):583–612. doi: 10.1111/tra.12163 (PMC4265843; doi:10.1111/tra.12163)
Supplement: Supplementary file 5 [file tra0015-0583-SD5.doc]

**Table S1.** *D^eff^_MACRO_* for Cy3-PEs in six cell lines, observed at a 33-ms resolution, complementing the results shown in Figs. 4, 6B, 10 and 11A.

| Cells | Lipids^a^ | *D^eff^_MACRO_* (median, µm^2^/s) | *D^eff^_MACRO_*  (mean ± SE, µm^2^/s) | Temp (˚C) | # of Examined Molecules |
| --- | --- | --- | --- | --- | --- |
| PtK2 | O | 0.33 | 0.32 ± 0.03 | 37 | 163 |
| COS-7 | O | 0.35 | 0.37 ± 0.03 | 37 | 45 |
| HASM | O | 0.17 | 0.19 ± 0.01 | 24 | 300 |
|  | M | 0.19 | 0.21 ± 0.01 | 24 | 318 |
|  | P | 0.22 | 0.26 ± 0.01 | 24 | 255 |
|  | O | 0.37 | 0.44 ± 0.02 | 37 | 260 |
|  | M | 0.38 | 0.45 ± 0.02 | 37 | 288 |
|  | P | 0.40 | 0.42 ± 0.01 | 37 | 589 |
| NRK | O | 0.39 | 0.38 ± 0.03 | 37 | 101 |
|  | M | 0.38 | 0.40 ± 0.02 | 37 | 162 |
|  | P | 0.41 | 0.44 ± 0.03 | 37 | 144 |
| T24 | O | 0.21 | 0.27 ± 0.02 | 37 | 114 |
|  | M | 0.24 | 0.27 ± 0.02 | 37 | 91 |
|  | P | 0.26 | 0.32 ± 0.02 | 37 | 120 |
| BHK | O | 0.33 | 0.33 ± 0.02 | 37 | 95 |
|  | M | 0.36 | 0.38 ± 0.03 | 37 | 104 |
|  | P | 0.34 | 0.44 ± 0.02 | 37 | 71 |

^a^O, Cy3-DOPE; M, Cy3-DMPE; P, Cy3-DPPE. In all of the cases shown here, 80~94% of the trajectories were classified into the simple-Brownian diffusion mode.
